# In vivo human keyhole limpet hemocyanin challenge in early phase drug development: A systematic review

**DOI:** 10.1111/cts.13457

**Published:** 2022-11-24

**Authors:** Philip G. Drennan, Dimitrios Karponis, Duncan Richards, Mark Coles, James N. Fullerton

**Affiliations:** ^1^ Kennedy Institute of Rheumatology, Nuffield Department of Orthopaedics, Rheumatology and Musculoskeletal Medicine University of Oxford Oxford UK; ^2^ Oxford University Hospitals NHS Foundation Trust Oxford UK; ^3^ Botnar Research Centre, Nuffield Department of Orthopaedics, Rheumatology and Musculoskeletal Medicine University of Oxford Oxford UK

## Abstract

Experimental exposure of healthy volunteers to the T‐cell dependent neoantigen keyhole limpet hemocyanin (KLH) permits the evaluation of immunomodulatory investigational medicinal product (IMP) pharmacology prior to the recruitment of patient populations. Despite widespread use, no standardized approach to the design and conduct of such studies has been agreed. The objective of this systematic review was to survey the published literature where KLH was used as a challenge agent, describing methodology, therapeutic targets addressed, and pharmacodynamic outcome measures. We searched MEDLINE, EMBASE, clinicaltrials.gov, and Cochrane CENTRAL for studies using KLH challenge in humans between January 1, 1994, and April 1, 2022. We described key study features, including KLH formulation, dose, use of adjuvants, route of administration, co‐administered IMPs, and end points. Of 2421 titles and abstracts screened, 46 met the inclusion criteria, including 14 (31%) early phase trials of IMP, of which 10 (71%) targeted T‐cell co‐stimulation. IMPs with diverse mechanisms demonstrated modulation of the humoral response to KLH, suggesting limited specificity of this end point. Two early phase IMP studies (14%) described the response to intradermal re‐challenge (delayed type hypersensitivity). Challenge regimens for IMP assessment were often incompletely described, and exhibited marked heterogeneity, including primary KLH dose (25‐fold variation: 100–2500 mcg), KLH formulation, and co‐administration with adjuvants. Methodological heterogeneity and failure to exploit the access to tissue‐level mechanism‐relevant end points afforded by KLH challenge has impaired the translational utility of this paradigm to date. Future standardization, characterization, and methodological development is required to permit tailored, appropriately powered, mechanism‐dependent study design to optimize drug development decisions.

## INTRODUCTION

### Immune challenge studies in drug development

The price of new medicines is driven to a large extent by high drug development costs, currently estimated at US $2.7 billion per new immunomodulatory drug approved by the US Food and Drug Administration.^1^ Failure of investigational medicinal products (IMPs) during development contributes an estimated 60% of this expense, with failure predominantly (60%–80%) due to inadequate efficacy.^2^, ^3^, ^4^ Recent data indicate the overall probability of success in phase II is ~25%: the lowest success rate of any stage in the clinical development pathway.^5^ Addressing phase II performance therefore affords the greatest opportunity to improve the overall probability of success, and may be facilitated by improved decision making in early‐phase development.

Processes associated with better decision making in early phase drug development have been discussed in the literature: variably described in AstraZeneca's “5R Framework,”^6^ Pfizer's “3‐Pillar” approach,^4^, ^7^ Merck's “Translational Medicines Guide,”^8^ and Eli Lilly's “Chorus initiative.”^9^ A recurring theme is the need to demonstrate proof of mechanism (PoM) via describing the pharmacokinetic/pharmacodynamic (PK/PD) relationship (exposure, target binding, and functional activity) at the biophase (target tissue) in humans prior to phase II commencement. The effect of a PoM strategy is potentially profound: in a recent analysis of the AstraZeneca pipeline, where PoM was established by end‐phase I, phase II success was 29%, versus 0% where it was not established.^4^ It follows that well‐characterized paradigms to determine PoM of immunomodulatory drugs in healthy volunteers are clearly needed.

One widely used method for interrogating the mechanisms of immunomodulatory drugs in vivo is experimental human immune challenge (HIC). In HIC, exogenous stimulants are administered to elicit activation of pathways, cell populations, and genes which are quiescent during homeostasis. In the context of therapeutic development, these may represent druggable targets or biologically relevant PD end points, whose modification by investigational medicinal products (IMPs) can be rapidly and thoroughly assessed in early phase clinical trials recruiting small numbers of participants. HIC paradigms can therefore be used to demonstrate PoM, confirm prior in vitro and animal data, and contribute to the determination of dose, population, and end point selection for subsequent clinical trials in patient cohorts. From a discovery perspective, HIC offers a powerful window into human immunology, and thus can maximize the scientific value of early phase clinical trials.^10^, ^11^, ^12^ Their utility is, however, predicated on the existence of broadly applicable standardized techniques with known, disease‐relevant response characteristics.

One key HIC model is the T‐cell dependent antigen response (TDAR), which may be followed by subsequent intradermal rechallenge and assessment of delayed type hypersensitivity (DTH). Use of a neoantigen (i.e., an antigen to which humans are typically immunologically naïve) for HIC‐TDAR, followed by rechallenge and DTH, allows end‐to‐end interrogation of innate and adaptive primary and memory responses occurring in accessible tissues (e.g., blood, skin, and lymph nodes). The controlled setting of HIC allows exploration and standardization of key experimental variables, such as primary antigen dose, timing of rechallenge relative to primary antigen exposure, and timing of outcome assessments.

Whereas many antigens may be used in the conduct of HIC studies, the xenogenic neoantigen keyhole limpet hemocyanin (KLH; derived from the Grand Keyhole Limpet *Megathura crenulata*) is considered a “model antigen” for this purpose.^13^ Because humans are commonly naïve to KLH epitopes prior to immunization (albeit with cross‐reactive responses to structurally similar endogenous or exogenous carbohydrate epitopes in some cases^14^, ^15^), KLH has advantages over alternate natural or therapeutically used antigens commonly used for HIC (e.g., varicella zoster or BaCG) which do not allow control of the degree, duration, and time‐elapsed since primary exposure. KLH has been used for over 50 years for TDAR/DTH assessment in both HIC and preclinical contexts.^16^, ^17^, ^18^, ^19^ Longstanding use has affirmed its utility as a safe and reliable model inducer of a TDAR/DTH response. However, despite widespread use in interventional and observational studies, HIC using KLH (“KLH challenge”) has historically lacked fundamental characterization and standardization.^13^, ^15^ For example, early KLH preparation methods yielded a product which contained substantial impurities, which could result in unpredictable effects on the immune response, and deleterious impacts on internal and external study validity. KLH is now available as a highly purified, good manufacturing practice (GMP)‐grade product, as either a “native” or high molecular weight (HMW) form, or a “subunit” form consisting of disaggregated subunits (designated KLH1 and KLH2) of ~400 kDa each.^15^ Although early studies have explored the dose–response of KLH neoantigen and DTH responses, to our knowledge, no studies in the modern era have systematically explored antigen dose–response using GMP‐grade (subunit or HMW) KLH.^20^, ^21^, ^22^, ^23^

Given the potential value of KLH challenge in immunomodulatory drug development, and previous concerns regarding its optimization, we sought to describe the current status of KLH challenge in this context. The aim of this review was therefore to systematically identify, collate, and describe HIC studies using KLH. We sought to (i) identify studies applying KLH challenge, focusing on application to early phase clinical trials, and related studies which may inform the optimization of the model; (ii) describe applications of human KLH challenge in terms of KLH formulation, use of adjuvants, dose regimen, outcomes assessed, and concordance of outcomes with preclinical immune challenge studies (using KLH and other T‐cell dependent antigens); and (iii) identify opportunities for further refinement of the model, in order to maximize the utility of this approach in early phase clinical trials of immunomodulatory IMP.

## METHODS

We used the Preferred Reporting Items for Systematic Reviews and Meta‐Analyses (PRISMA) framework to structure this review (Table S1‐S5),^24^ and managed the review process using the Rayyan online systematic review platform.^25^

### Search strategy

We identified studies conducted between January 1, 1994, and April 1, 2022, where KLH was administered to humans via percutaneous injection to elicit an immune response for nontherapeutic purposes.

We searched the following databases and registries: (i) Medline (Ovid), (ii) EMBASE (Ovid), (iii) clinicaltrials.gov, and (iv) Cochrane CENTRAL trials register. The full electronic search strategy is available in Table S2. Reference lists of included studies and previous relevant reviews were also searched.^13^, ^15^ Initial screening of titles and abstracts was performed by a single reviewer, followed by independent review of potentially relevant manuscripts by two reviewers. Any conflicts were resolved following review of the manuscript and discussion between the reviewers. Following Swaminathan et al.^13^ we restricted our search to studies published after 1994, due to the significant uncertainty associated with generalization of earlier studies using poorly purified, non‐GMP grade formulations of KLH.

### Inclusion and exclusion criteria

We included published studies where KLH was administered to healthy volunteers or patient populations via percutaneous routes (i.e., intramuscular, subcutaneous, or intradermal injections). The following studies were excluded: (i) studies where KLH was administered as a vaccine adjuvant or control substance in cancer vaccine studies, (ii) in vitro or ex vivo analyses of human tissue or cells where KLH was not administered to participants prior to sampling, (iii) studies where KLH was administered as part of a therapeutic regimen (e.g., bladder cancer), (iv) studies where KLH was given by nonpercutaneous routes (e.g., enteral, inhalational), and (v) studies reported as conference abstracts only.

### Reporting quality

We assessed reporting quality using the Template for Intervention Description and Replication (TIDieR) checklist, adapted to capture key aspects relevant to the reproducible conduct of KLH challenge (Table S3).^26^ Given the intended focus of the review, the reporting quality assessment was restricted to the studies applying KLH challenge to early phase clinical trials. Appraisals of the two independent reviews were compared and any disagreements resolved through discussion and consensus, or by third party adjudication if necessary.

### Data extraction

We categorized identified studies into: (i) early phase (I or II) clinical trials, (ii) late phase clinical trials, postmarketing surveillance, or observational studies of immunomodulatory drugs, (iii) other contexts (e.g., exploration of fundamental immunology in healthy participants, effect of a disease state, or effect of a non‐pharmacological exposure or intervention).

Variables extracted from each study included date of publication, study aim, exposures (both pharmacological and non‐pharmacological), KLH regimen (product, source, formulation, use of adjuvant, dose, and timing of rechallenge dose[s]), and outcomes assessed (KLH‐specific and nonspecific PD end points). For IMPs, the rationale for a specific KLH challenge regimen (e.g., KLH dose, timing relative to the IMP) and evidence of modulation of KLH‐specific PD end points or PoM was described, where reported. Finally, to further understand the translational relevance of the KLH challenge approach to early phase studies of IMP, where a corresponding T‐cell dependent antigen challenge‐drug combination was reported by the study authors, we evaluated the concordance of the findings between the preclinical and clinical studies.

## RESULTS

### Human KLH challenge applications: 1994–2022

**FIGURE 1:**
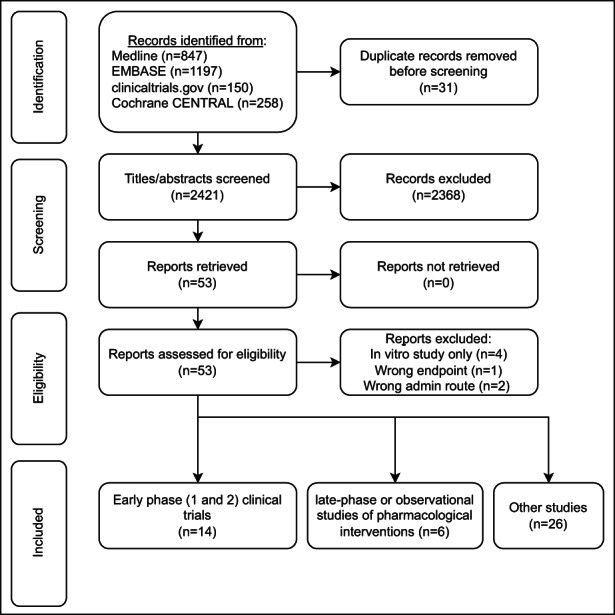
Preferred reporting items for systematic reviews and meta‐analyses (PRISMA) flow diagram describing systematic literature search for experimental human immune challenge studies using keyhole limpet hemocyanin

**FIGURE 2:**
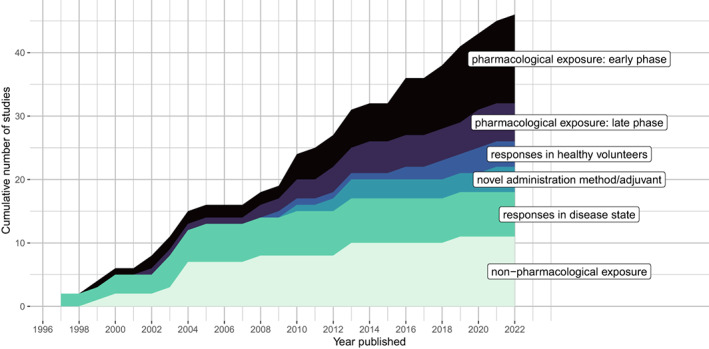
Experimental human immune challenge studies using keyhole limpet hemocyanin published between January 1, 1994, April 1, 2022. *x*‐axis: year published, *y*‐axis, cumulative number of studies within each application category

We identified 46 studies published between 1994 and 2022 which met the inclusion criteria (Figure 1). Fourteen studies (30%) described applications to early phase drug development (Table 1), and six (13%) described applications to late phase clinical trials, postmarketing surveillance, or observational studies of immunomodulatory drugs (Table 2). Twenty‐six studies (57%) described application of the model for other purposes, including five (11%) describing responses in healthy volunteers (including model optimization prior to intended application in a clinical trial^27^, ^28^, ^29^), 11 (24%) describing effects of non‐pharmacological exposures, seven (15%) describing effects of disease states, two (4%) describing development of novel assays of KLH‐specific responses, and one (2%) describing the assessment of a novel adjuvant (Table S4). There was increasing application of KLH challenge for the assessment of immunomodulatory drugs over the review period (Figure 2): between 1994 and 2008, four studies described KLH challenge in the context of pharmacological exposure (including 2 early phase clinical trials), compared to 16 studies between 2009 and 2022 (12 early phase trials).

**TABLE 1:** Early phase (I and II) clinical trials (*n* = 14) of investigational medicinal products using KLH challenge

Study (year published), phase	Drug: mechanism	Study population (number)	KLH regimen: formulation (source), dose, route, site, timing	Antibody assessment (method, timepoint)	Other KLH‐specific PD end points	Non‐KLH specific PD end points	Evidence for modulation of KLH‐specific end points/proof‐of‐mechanism and reported concordance with animal studies
Abrams (1999),^30^ phase I	Abatacept: CTLA4‐Ig fusion protein, antagonist of the CD28‐CD80/86 interaction	Patients with psoriasis (*n* = 43)	HMW‐KLH (Intracel), 1000 μg ID^a^, administered 14 days before and 29 days after initiation of abatacept or control	Anti‐KLH Ig total (ELISA), measured 14 and 28 days after second KLH immunization	Nil	Bacteriophage φX174 neoantigen response Psoriasis disease activity Serial histological response (histology and immunohistochemistry) of psoriatic lesions (including epidermal thickness and T cell enumeration) Blood lymphocyte subpopulations	Suppression of antibody titers (relative to control cohort) observed in one or more patients accrued to all dose levels. Normal peak antibody titers were observed in some patients in the highest dose cohort. Qualitatively similar observations in preclinical models with CTLA4Ig inhibiting T cell mediated antibody response in mice receiving KLH and SRBC challenge, and antibody responses in monkeys receiving an unspecified antigen.^78^, ^79^ In the murine study, CTLA4Ig was more effective in suppressing primary antibody responses to KLH than secondary responses.
Van der Kolk (2002),^36^ phase I/II (new indication post‐initial marketing authorization)	Rituximab: chimeric anti‐CD20 mAb	Patients with relapsed low‐grade lymphoma (*n* = 11 total, *n* = 7 received KLH challenge)	HMW‐KLH (Calbiochem) 1000 μg s.c., either 14 days before, or 14 days after rituximab treatment	Anti‐KLH IgG (ELISA) assessed 14 days post‐KLH	Nil	Antibody responses to hepatitis A vaccine, tetanus toxoid, and poliomyelitis vaccine. Total immunoglobulin concentrations. Peripheral B cell count.	Nil: None of the patients developed anti‐KLH IgG responses before or after rituximab treatment.
Bingham (2010),^31^ phase II	Rituximab	Patients with rheumatoid arthritis receiving methotrexate (*n* = 103)	HMW‐KLH (Intracel) 1000 μg s.c., 36 weeks post‐rituximab course (fortnightly ×2 doses)	Anti‐KLH IgG at baseline and 28 days post‐KLH	Nil	Antibody responses to pneumococcal polysaccharide antigen and tetanus toxoid. ID candida antigen DTH response. Total immunoglobulin levels (IgM, IgG, IgG subsets, IgA). Lymphocyte subsets.	Proportion of patients mounting a quantifiable anti‐KLH IgG response was lower in the rituximab‐treated group (30 of 64 patients [47%]) than in the MTX alone–treated group (25 of 27 patients [93%]), with ~3× lower geometric mean titers in the rituximab group. Qualitatively similar suppression of antibody responses to a KLH‐hapten conjugate in baboons and mice exposed to anti‐CD20 antibodies^80^, ^81^
Smith (2010),^35^ phase II	Recombinant human HGH (Somatotrophin)	Patients with HIV infection (*n* = 60)	Subunit KLH (Immucothel), 100 μg ID, 16 and 20 weeks post‐initiation of rhGH or standard care	Nil	Lymphoproliferative responses to in vitro KLH stimulation of PBMCs	HIV‐1 viral load, T‐lymphocyte subsets, PBMC lymphoproliferative assays (pokeweed mitogen, candida, HIV p24 antigen), T cell receptor circles, thymus size on CT scan	Two of the participants in the intervention arm (*n* = 30), and no participants in the control arm (*n* = 30) developed new lymphoproliferative responses to KLH at week 24.
Jain (2011),^34^ phase II	Recombinant CD40L	Pediatric patients with X‐linked hyper IgM syndrome (*n* = 3)	KLH (formulation, source and route not stated) 2500 μg, 8, 12, and 16 weeks after initiation of regular rCD40L. Three subsequent ID KLH challenges (dose not stated)	Anti KLH IgG (ELISA)	DTH response (induration)	DTH response to recall antigens (candida, mumps and purified protein derivative). Serum total immunoglobulins. Antibody response to φX‐174. Cytokine release (IFN‐γ and TNF‐α). PBMC stimulation (anti‐CD3, SEA, SEB). B cell mutation analysis. CT imaging of axillary lymph nodes. Histology on lymph node biopsy.	All 3 participants developed a positive DTH response following 10–20 weeks of rCD40L treatment, followed by negative responses after a 12‐week drug free interval, and return of positive DTH responses during subsequent rCD40L treatment. No patients developed detectable anti‐KLH IgG.
Curti (2013),^37^ phase I	MEDI6469/9B12: OX40 agonist	Patients with advanced solid malignancies (*n* = 30)	Subunit KLH (Immucothel) s.c. (dose not stated). Arm A: KLH day 1, arm B: day 29, relative to anti‐OX40 initiation	Anti‐KLH IgG (ELISA) assessed day 15 (arm A) or day 43 (arm B), i.e., 14 days after KLH dose.	Nil	Anti‐tetanus antibody. Blood lymphocyte subpopulations. Tumor‐specific T cell assays. T cell proliferation (tetanus toxoid, anti‐CD28/anti‐CD49d). Radiological tumor response.	A greater increase in KLH (and tetanus) antibody titer (assessed D + 15 post‐KLH) was observed in patients immunized on the same day as anti‐OX40 compared to those immunized 28 days later. Qualitatively similar effects seen in rhesus macaques exposed to OX40 agonists immunized with simian immunodeficiency virus protein gp130, with higher anti‐gp130 antibody titers in the exposed group.^82^
Poirier (2016),^41^ phase I	FR104: pegylated anti‐CD28 monovalent Fab’ antibody (antagonist)	Healthy volunteers (*n* = 64 total, *n* = 33 in KLH challenge cohorts)	KLH (formulation, source, route, dose not stated—likely Immucothel), presumed given on same day as FR104 (SAD cohorts)	Anti‐KLH IgG (ELISA) assessed at screening, days 15, 29, 57, 85, and/or 113	Nil	Serum cytokine concentration (safety end point). CD28 receptor occupancy. T lymphocyte subsets in blood (flow cytometry). Ex vivo SEB and LPS stimulation, pre/post dose (TruCulture system) with cytokine release (IL‐2, IL‐8, IFNγ) quantification. EBV reactivation.	Dose‐dependent inhibition of time to detection of anti‐KLH IgG.
Shi (2016),^42^ phase I	Lulizumab pegol (BMS‐931699: pegylated monovalent anti‐CD28 domain antibody (antagonist)	Healthy volunteers (*n* = 16)	Subunit KLH (Immucothel) 1000 μg s.c. given on same day as lulizumab	Anti‐KLH IgM and IgG (ELISA), assessed days 1, 8, 15, and 29	Nil	Serum cytokine concentration (safety end point). Lymphocyte subsets. Receptor occupancy on CD4+ and CD8+ CD11a^low^ T lymphocytes (a subpopulation of CD8+ T cells shown to uniformly express CD28).	Higher dose levels of lulizumab resulted in inhibition of anti‐KLH IgG titers for ≥2 weeks postdose, with suppression of antibody responses associated with >80% CD28 receptor occupancy. Model based approaches used to demonstrate quantitatively similar effects of lulizumab in monkeys on KLH IgG suppression, based on degree of anti CD28 receptor occupancy.^46^
Sullivan (2016),^32^ phase I	Prezalumab (AMG557): human ICOSL mAb (antagonist)	Patients with SLE (*n* = 112)	Sub‐unit KLH (Vacmune) 1000 μg ID D2 or 8 and D29 or 36 post prezalumab (depending on cohort) in SAD cohorts. 1000 μg ID D57 and 85 post initiation of prezalumab in MAD cohorts.	Anti‐KLH IgM and IgG (flow cytometric bead array)	Nil	ICOSL target occupancy, total and free ICOSL levels on B cells. Tetanus antitoxin IgG, total IgG. Blood lymphocyte subpopulations. SLE disease activity scores and biomarkers.	Prezalumab in MAD cohorts resulted in decreased anti‐KLH IgG, for example, placebo, with no change in the SAD cohort. Evidence of dose response with higher prezalumab exposure in participants without detectable pre‐existing anti‐KLH IgG titers. Inflection point in IgG suppression correlated with steady‐state trough prezalumab concentrations above the IC_99_ target for receptor occupancy. Anti‐KLH IgM was not reduced compared to placebo in any cohort. Qualitatively similar effect of pharmacological ISCOL‐B7‐related protein 1 blockade seen in mice challenged with KLH, with reduction in anti‐KLH IgG and absence of effect on anti‐KLH IgM^83^
St. Clair (2018),^33^ phase II	Baminercept: lymphotoxin‐β receptor IgG fusion protein	Patients with Sjogren's syndrome (*n* = 52)	Subunit KLH (Immucothel) 1000 μg s.c. with Montanide ISA‐51 VG 8 weeks post‐initiation of weekly baminercept	Anti‐KLH IgM and IgG (ELISA), assessed day 28 post‐KLH	Nil	Pneumococcal polysaccharide vaccine antibody response. Sjögren's syndrome clinical end points and autoantibodies, cytokines, and chemokines. B and T cell subsets, including circulating memory T cells.	Baminercept did not suppress the anti‐KLH IgG response to KLH. An increase in anti‐KLH IgM with baminercept treatment was reported, thought to be consistent with impaired class switch recombination. Authors report findings consistent with previous rodent studies demonstrating impaired class switch recombination in the presence of baminercept (study not referenced)
Karnell (2019),^43^ phase I	Dazodalibep (VIB4920/MEDI4920/HZN4920): CD40L binding protein	Healthy volunteers (*n* = 56)^b^	KLH (formulation and source not stated) plus aluminium hydroxide 1000 μg s.c. 14 days before and 15 days post‐dazodalibep	Anti‐KLH IgG (ELISA) days 22, 29, 43, 57, 85, and 113 post‐dazodalibep	Nil	sCD40L as a measure of target engagement	Dazodalibep inhibited anti‐KLH IgG in a dose‐dependent fashion, with no inhibition at low doses, and near complete suppression of responses at the highest dose level. Qualitatively similar responses in mice immunized with SRBC in the presence of an inhibitor of an analogous T cell co‐stimulation interaction, with suppression of anti‐SRBC IgG response commensurate with inhibition of the germinal center B cell response in a dose‐dependent fashion.^43^
Espié (2019),^47^ phase I	Iscalimab (CFZ533): fully human, aglycosylated IgG_1_, anti‐CD40.	Healthy volunteers (*n* = 36)	KLH (formulation and source not stated) 115 μg i.m. day 3 post‐iscalimab, second KLH dose at varying timepoints according to cohort, corresponding to expected loss of CD40 receptor occupancy (days 29, 43, 57, 71, 82, and 85)	Anti‐KLH IgM and IgG assessed at screening or day −1, and days 8, 15, and 22 post‐first KLH dose, and ~14, 28, and 42 post‐second KLH dose.	Nil	Free and total CD40 receptor levels (percent change from baseline) to assess extent and duration of target engagement on whole blood B cells. Ex vivo stimulation of whole blood with CD154, with assessment of B cell activation by measuring CD69 and CD23 expression on CD19‐positive B cells by flow cytometry. EBV and CMV reactivation. Serum cytokine concentration (safety end point).	Higher doses of iscalimab transiently suppressed anti‐KLH responses following immunization for ~3–4 weeks. Suppression observed for lower doses for a reduced duration. Delayed anti‐KLH primary responses were subsequently observed in all subjects (reduced c.f. placebo control), corresponding to declining iscalimab concentrations and CD40 occupancy. All subjects mounted responses to the rechallenge KLH dose (given after predicted loss of receptor occupancy). Qualitatively similar results seen in nonhuman primates (*n* = 4) exposed to CFZ533 and immunized with KLH in alum.^84^ Although limited by small numbers, this study suggested dose‐dependent effects of CFZ533 on anti‐KLH IgG response and germinal center formation.
Yang (2021),^45^ phase I	Acazicolcept (ALPN‐101): ICOSL variant Ig Fc fusion protein, dual ICOS/CD28 antagonist.	Healthy volunteers (*n* = 92 total, *n* = 36 in KLH challenge cohorts)	KLH (Stellar biotechnologies, formulation not stated) 1000 μg s.c. day 0 or 1 post‐acazicolcept administration	Anti‐KLH IgM and IgG (ELISA) assessed days 0, 7, 14, 21, and 28 in SAD cohort, and days 0, 7, 14, 21, 28, 35, 42, and 49 in MAD cohort.	Nil	Serum cytokine concentration (safety end point). CD4/CD8 T cell target saturation (CD28 and ICOS). Blood lymphocyte subpopulations. Ex vivo SEB stimulation, pre/post dose (TruCulture system) with cytokine release (IL‐2, IL‐6, IFNγ, TNFα) quantification.	Single dose and repeated doses of acazicolcept resulted in significant reductions in anti‐KLH antibody titers compared to placebo groups. Trend toward dose‐dependent inhibition in SAD cohort, especially at day 28. Qualitatively similar effects of acazicolcept seen in a mouse model of collagen induced arthritis, with reduced anti‐collagen antibodies, paw inflammation, and serum cytokine concentrations,^85^ and in studies of mice challenged with KLH and SRBC, with reduced antibody titers, germinal center formation, and T follicular helper cells.^86^
Saghari (2022),^38^ phase I	KY1005: OX40L antagonist	Healthy volunteers (*n* = 64)	Subunit KLH (Immucothel) 100 μg with aluminium hydroxide 900 μg i.m., day 7 post‐third dose of KY1005 (administered 0, 4, and 8 weeks). ID rechallenge KLH 1 μg day 21 post‐primary KLH dose.	Anti‐KLH IgM and IgG (ELISA) assessed day 21 post‐primary KLH immunization	DTH response using clinical measurement (induration and erythema) and imaging techniques	OX40 and OX40L expression on PBMCs (flow cytometry). Anti‐tetanus toxoid antibody titer.	Anti KLH IgM and IgG antibody titer measured using ELISA: no statistically significant difference in antibody titers across dose levels. Post hoc analysis suggesting modest exposure (AUC) ‐response (IgM and IgG) relationship. Cutaneous blood perfusion with laser speckle contrast imaging: Reduction of cutaneous blood perfusion measurements in KY1005 groups compared to placebo without clear dose–response relationship demonstrated. Skin erythema measured using multispectral imaging: evidence of reduced skin erythema measurements compared to placebo. Maximum KY1005 dose in this study selected based on equivalent predicted exposure associated with maximum anti‐KLH IgG suppression in monkeys challenged with KLH, using a model‐based approach (study not referenced).

Abbreviations: AUC, area under the curve; CT, computed tomography; CMV, cytomegalovirus; DTH, delayed type hypersensitivity; EBV, Epstein–Barr virus; ELISA, enzyme‐linked immunosorbent assay; KLH, keyhole limpet hemocyanin; mAb, monoclonal antibody; MAD, multiple ascending dose; MTX, methotrexate; PBMC, peripheral blood mononuclear cell; PD, pharmacodynamic; rhGH, recombinant human growth hormone; SAD, single ascending dose; SLE, systemic lupus erythematosus.

^a^ Participants (*n* = 4) in the lowest dose cohort (0.5 mg/kg) received 100 μg KLH. b

**TABLE 2:** Late phase and post‐marketing trials (*n* = 6) investigating drug effects on response to KLH challenge

Study (year published), phase	Drug: mechanism	Study population (number)	KLH regimen: formulation (source), dose, route, site, timing	Antibody assessment (method), timepoint)	Other KLH‐specific PD endpoints	Non‐KLH specific PD endpoints	Evidence for modulation of KLH‐specific endpoints
Rentenaar (2002),^51^ observational	Three cohorts: (i) prednisolone (P)/cyclosporine A (CsA); (ii) P/CsA/anti CD3 mAb; (iii) P/CsA/ MMF	Patients with renal transplant (*n* = 34)	HMW‐KLH (Calbiochem) 1000 μg s.c.	Anti‐KLH IgG (ELISA), assessed day 14 post‐KLH, rechallenge dose 100 μg ID day 14	DTH response: induration diameter, cell surface marker expression on skin biopsy (CD3, ICAM‐1, VCAM‐1, E‐selectin)	In vitro T cell proliferation	Significant reduction in anti‐KLH IgG responses in renal transplant group c.f. healthy volunteers. All healthy volunteers developed DTH responses. None of the renal transplant patients receiving IgA CD3 mAb rejection treatment in addition to P/CsA, and only two of the patients from the P/CsA and P/CsA/MMF groups had a positive DTH response.
Saville (2008),^49^ phase IV/post‐marketing surveillance	Rituximab	Healthy volunteers (*n* = 84). Patients with NHL (*n* = 110)	KLH (formulation, source, dose, route not stated), 36 weeks post‐rituximab course (weekly ×4 doses), or immediately in healthy volunteers	Anti‐KLH IgG (method not stated), assessed day 28 post KLH	Nil	Pre‐existing bacterial and viral antibody titers. Antibody response to tetanus toxoid	4 of 108 (4%) participants in the NHL group had a doubling of anti‐KLH antibody titer, compared to 58 of 84 (69%) healthy volunteers
Struijk (2010),^53^ observational study	Three cohorts: (i) P/CsA, (ii) P/mycophenolic acid, (iii) P/everolimus, (iv) healthy volunteers	Healthy volunteers (*n* = 13). Patients with renal transplant (*n* = 36)	Subunit KLH (Immucothel) 1000 μg s.c.	Anti‐KLH IgG (ELISA) day 14 post‐KLH	Nil	Antibody responses to pneumococcal polysaccharide antigen and tetanus toxoid. Tetanus toxoid ELISPOT assay (IL‐2, IFN‐γ, IL‐4).	Primary anti‐KLH IgG response significantly lower in all patient groups compared to healthy volunteers. Some evidence of increased anti‐KLH IgG response in everolimus cohort c.f. other immunosuppressive regimens
Boulton (2012)^50^	Fingolimod: sphinosine‐1‐phosphate receptor modulator	Healthy volunteers (*n* = 72)	Subunit KLH (Immucothel) 100 μg plus aluminium hydroxide (dose not stated) i.m. on days 7, 14, 21 post‐initiation of regular fingolimod (or placebo). ID rechallenge KLH 10 μg day 28 post first KLH dose	Anti‐KLH IgM and IgG assessed at baseline, days 7, 14, 21, 28, 42, and 56	DTH response	Antibody responses to pneumococcal polysaccharide antigen and tetanus toxoid. DTH response to tetanus toxoid and candida antigen. Lymphocyte subsets	Primary anti‐KLH and IgM and IgG response lower in fingolimod groups. Some evidence of dose response with lower anti‐KLH IgG in high vs. low fingolimod dose levels
Kaufman (2014),^52^ phase IV/post‐marketing surveillance	Natalizumab: humanized IgG4k mAb, a4‐integrin antagonist	Patients with multiple sclerosis (*n* = 60)	Subunit KLH (Immucothel) 1000 μg s.c. on days 0, 14, and 28, either 2 months before or 6 months after natalizumab	Anti‐KLH IgG assessed days 14, 28, 56	Nil	Antibody response to tetanus toxoid	Natalizumab had no discernible effect on anti‐KLH IgG responses
Bar‐Or (2020),^48^ phase IIIb	Ocrelizumab: humanized anti‐CD20 mAb	Patients with multiple sclerosis (*n* = 102)	KLH (formulation, source, not stated) 1000 μg i.m. days 0, 28, and 56. For ocrelizumab group, KLH commenced 12 weeks postdose (fortnightly ×2 doses)	Anti‐KLH IgM, IgG, assessed days 28, 56, and 84 post‐KLH	Nil	Antibody responses to pneumococcal polysaccharide antigen, pneumococcal conjugate vaccine, influenza vaccine, and tetanus toxoid. Peripheral blood B cell counts. Total immunoglobulin concentrations	Anti‐KLH IgM and IgG titers significantly reduced in ocrelizumab group c.f. control at 4, 8, and 12 weeks

Abbreviations: DTH, delayed type hypersensitivity; ELISA, enzyme‐linked immunosorbent assay; KLH, keyhole limpet hemocyanin; mAb, monoclonal antibody; MMF, mycophenolate mofetil; NHL, non‐Hodgkin's lymphoma.

### Early phase (I or II) clinical trials

Of the 14 early phase clinical trials identified, six (43%) recruited healthy volunteers, and eight (57%) recruited patients with autoimmune conditions (psoriasis,^30^ rheumatoid arthritis,^31^ systemic lupus erythematosus,^32^ and Sjogren's syndrome^33^), inherited immunodeficiencies,^34^ HIV,^35^ or malignancy.^36^

#### KLH challenge regimens

GMP‐grade, subunit KLH was used in six of 14 studies (43%, all from a single supplier: Biosyn), which was administered with an adjuvant in three studies (aluminium hydroxide or Montanide ISA‐51). HMW‐KLH was used in three studies (21%), and an unspecified formulation in five studies (36%). The most common primary KLH dose used was 1000 μg (9 studies, range: 100–2500 μg), and was administered by the subcutaneous route in seven studies, intradermal route in two studies, intramuscular route in two studies, and via an unspecified route in two studies. Seven studies administered a single dose of KLH, whereas seven studies administered more than one dose. In two studies, intradermal KLH was administered as a rechallenge followed by DTH assessment (see outcome assessment below). The primary dose of KLH was administered prior to the IMP in two studies, concurrently with initiation of the IMP in three studies, and following initiation of the IMP in seven studies. One study compared responses to KLH according to administration 14 days before or 14 days after the initiation of the IMP (rituximab^36^), whereas another compared responses to KLH when administered concurrently to or 29 days following initiation of the IMP (an OX40 agonist).^37^

#### Outcome assessment and effect of study drug on KLH immune response

The response to KLH challenge was a primary outcome in two of the 14 (14%) studies identified, and a secondary or exploratory outcome in 12 studies (86%), which, consistent with their early phase status, generally focused on safety, PK, and efficacy biomarkers. Thirteen studies (93%) reported humoral responses to KLH, typically using enzyme‐linked immunosorbent assay with one study using a flow‐cytometric bead‐based assay.^32^ Seven studies reported anti‐KLH IgM responses in addition to IgG. Studies varied in the timepoints of antibody assessments and frequently assessed responses at multiple timepoints, most commonly 14 and 28 days (range 7–113 days) post‐KLH administration.

Of the two studies which elicited DTH by intradermal KLH rechallenge, both assessed responses clinically, via measurement of erythema, and/or induration using the “ball‐point pen technique” commonly used for assessment of Mantoux/tuberculin skin tests.^34^, ^38^, ^39^ In addition, Saghari et al.^38^ used noninvasive imaging techniques to objectively quantify the cutaneous DTH response (erythema and induration), and assessed induration, erythema, tenderness, and pain using a validated toxicity grading scale.^38^, ^40^

#### Other outcome assessments

All studies incorporated KLH challenge into a larger battery of PD assessments. Most studies reported drug target occupancy (e.g., on a relevant T‐cell subset).^32^, ^37^, ^38^, ^41^, ^42^, ^43^, ^44^, ^45^ Downstream PD outcomes included serum cytokine concentrations (commonly as a safety end point in CD28‐targeting agents),^33^, ^41^, ^42^, ^44^, ^45^ blood lymphocyte subsets,^30^, ^32^, ^33^, ^37^, ^41^, ^42^ viral reactivation (Epstein–Barr virus [EBV] and cytomegalovirus [CMV]),^41^, ^44^ response to vaccination with other antigens (e.g., tetanus toxoid,^31^, ^36^, ^37^, ^38^ pneumococcal polysaccharide vaccine,^33^ and bacteriophage φX174^30^), and effect of the IMP on pre‐existing antibody titers (anti‐tetanus toxoid IgG).^32^ A smaller number of studies reported end points based on ex vivo assays, including cytokine release following antigen stimulation with presensitized antigens (tetanus^37^ and candida^35^) or nonspecific stimulation (e.g., SEB, LPS, and CD154).^34^, ^41^, ^44^, ^45^ Studies incorporating patient populations reported disease‐specific end points, including histological response (e.g., psoriatic plaques^30^ and lymph nodes^34^), and disease activity end points assessed clinically or radiologically (e.g., autoimmune disease activity scores,^30^, ^33^ tumor response,^37^ thymus size,^35^ and lymph node size^34^).

#### Immunomodulatory mechanisms and proof‐of‐mechanism

**FIGURE 3:**
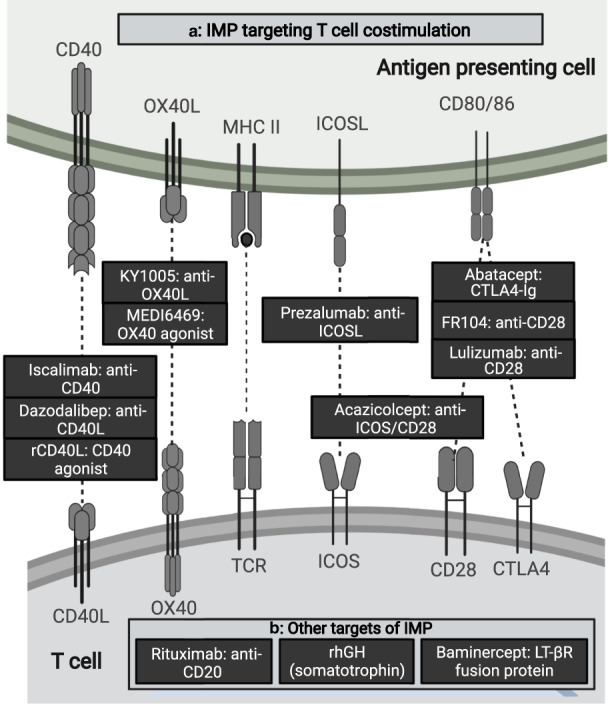
Molecular targets of early phase clinical trials of investigational medicinal products (IMPs) applying keyhole limpet hemocyanin (KLH) challenge 1994–2022. (a) IMP targeting T cell co‐stimulation (receptor interactions displayed graphically, top: antigen presenting cell, bottom: T cell), (b) other targets (listed). Created with BioRender.com

Eight of the 14 IMP (57%) studied in early phase clinical trials were inhibitors of T‐cell co‐stimulation (Figure 3), including CD28 (FR104 and lulizumab),^41^, ^42^ CD40 (dazodalibep),^43^, ^44^ OX40L (amlitelimab/KY1005),^38^ ISCOSL (prezalumab),^32^ dual ICOSL/CD28 (acazicolcept),^45^ and CD80/86 (abatacept).^30^ All but one of these studies demonstrated inhibition of anti‐KLH antibody responses relative to placebo, with a nonstatistically significant trend toward inhibition in the remaining study.^38^ Exposure‐response relationships were assessed more formally in some cases (e.g., by modeling antibody titers as a function of study drug exposure area under the curve [AUC] or receptor occupancy) these assessments generally demonstrated clearer associations with the PD response to KLH as compared to dose–response.^32^, ^38^, ^41^, ^43^

Five studies of immunosuppressive IMP were performed in patient populations (phase Ib or II), affording the potential for concurrent assessment of IMP modulation of both KLH response, and disease activity (or related biomarkers). Abrams et al.^30^ reported a study of abatacept (antagonist of the CD28‐CD80/86 interaction) in patients with psoriasis. In this study, suppression of total anti‐KLH antibody titers (compared to a control group) was seen in one or more patients at all dose levels 2 weeks after secondary immunization with 1000 μg HMW‐KLH, although no clear dose–response was seen. Conversely, dose‐dependent responses were seen in both clinical activity and histological markers of psoriatic activity, such as intralesional T cells. Sullivan et al.^32^ reported on the effects of AMG 557 (prezalumab), an ICOSL inhibitor, in an exploratory phase Ib study of patients with systemic lupus erythematosus (SLE) with inflammatory arthritis. AMG 557 treatment resulted in a significant reduction of anti‐KLH IgG responses following KLH challenge (1000 μg subunit KLH ID, 4 weeks apart). Notably, this effect was seen concurrently with acceptable target occupancy on circulating B cells (steady‐state trough concentrations above IC_99_), but with no observed effect of SLE‐related laboratory biomarkers (autoantibodies and complement concentrations) or disease activity scores. The authors asserted this lack of clinical response was consistent with the “mild, stable disease” status of the patients. St. Clair et al.^33^ reported a phase II study of baminercept (a lymphotoxin β receptor fusion protein) in patients with Sjogren's syndrome challenged with subunit KLH 1000 μg with Montanide ISA‐51. There was no evidence of suppression of anti‐KLH IgG responses in the intervention group, although mean anti‐KLH IgM responses were higher, compared with control (statistical significance not reported), postulated by the authors as possibly consistent with an effect on antibody class‐switching. No effect on disease activity was observed. Two studies reported antibody responses to HMW‐KLH in studies of the chimeric anti‐CD20 mAb rituximab, although no clinical correlates could be derived from these studies, with the KLH challenge regimen failing to elicit a response in either the control or intervention groups in one study of patients with relapsed low‐grade lymphoma,^36^ and another study where the focus was on response to immunizations (e.g., for understanding infection risk) and where no clinical responses were measured.^31^

Three studies reported application of KLH challenge in the context of (potentially) immunostimulatory drugs. Curti et al.^37^ conducted a noncontrolled phase I study of an investigational OX40 agonist in a heterogenous group of patients with metastatic solid tumors, and demonstrated a statistically significant increase in anti‐KLH IgG in the treatment groups. Regression of at least one metastatic lesion was reported in 12 of 30 (40% of) patients, although any potential correlation with response to KLH within individuals was not reported. Jain et al. described a small case series (*n* = 3) in which children with X‐linked hyper IgM syndrome (an immune deficiency disorder caused by mutations in the CD40 ligand gene) receiving treatment with recombinant CD40L (rCD40L) successfully mounted a KLH DTH response following study drug initiation, whereas no antibody responses to KLH were detected. Notably, in these patients, the DTH response on subsequent intradermal re‐challenge was absent after a drug‐free period, but returned following rCD40L re‐initiation.^34^ Finally, Smith et al.^35^ reported the use of recombinant human growth hormone (rhGH; Somatotrophin) in patients with advanced HIV infection, postulating potential beneficial effects on CD4^+^ T cell count and immune function, including response to KLH challenge. Overall, the effects of the intervention on the primary outcome were modest. No differences in in vitro lymphoproliferative responses to KLH were discerned in either the rhGH or control (antiretroviral therapy only) groups.

#### Concordance with preclinical immune challenge studies

Ten of 14 studies (71%) compared observed IMP effects on anti‐KLH antibody responses to those observed in animals receiving challenges with T‐cell dependent antigens (including KLH) and the same IMP, or a species‐specific analogue (Table 1). In all cases, the response to the IMP was qualitatively similar between the human and animal studies.

Two studies used model‐based approaches to bridge KLH challenge responses between species for the purposes of dose‐selection and/or exposure‐response analysis. Saghari et al.^38^ used PK considerations and allometric scaling to inform KY1005 dose selection in their (human) study, based on exposures observed to cause maximal suppression of anti KLH‐IgG in monkeys. In studies of a CD28 antagonist (lulizumab pegol), Shi et al. demonstrated that in both humans and monkeys, maximal suppression of anti‐KLH IgG response occurred with dose regimens where greater than 80% receptor occupancy was maintained for at least 2 weeks.^42^, ^46^

#### Reporting quality

Results of the adapted TIDieR checklist for assessment of reporting quality for the identified early phase trials are shown in Table S5. Whereas the rationale for using KLH challenge was explained in most studies (13/14, 93%), the rationale for key aspects of the challenge regimen (e.g., dose and use of adjuvants) was generally not. Two studies (14%) explicitly referenced previous studies to explain regimen decisions (i.e., justified KLH regimen on the basis of precedent).^36^, ^38^ One study timed KLH rechallenge to coincide with expected loss of receptor occupancy of the study drug at different dose levels.^47^ Other key aspects of KLH challenge regimens were frequently not described. For example, four studies (29%) failed to describe the formulation and supplier of the KLH administered. Two studies (14%) did not describe the route of KLH administration, whereas 12 studies (86%) did not describe the body site of KLH administration. One study did not describe the dose of KLH administered. Five studies (36%) did not refer to the conduct of KLH challenge in the study abstract.

### Late phase clinical trials, postmarketing surveillance, or observational studies of immunomodulatory drugs

Six of the 42 studies (14%) described application of KLH challenge to assess the activity of drugs in late‐phase clinical trials and observational studies. Collectively these studies attest to suppression of the humoral immune response to KLH by anti‐CD20 agents (rituximab and ocrelizumab),^48^, ^49^ S1PR modulation (fingolimod),^50^ IMPDH inhibition (mycophenolic acid),^51^ and lack of effect of an anti‐α4‐integrin mAb (natalizumab).^52^ In one observational study of patients who underwent renal transplantation receiving prednisolone and cyclosporine, the use of everolimus as a third agent was associated with retention of primary humoral immune response to KLH, in contrast to complete suppression of response seen with mycophenolic acid as a third agent.^53^

Two studies in this category reported assessment of DTH as an end point. In one study, healthy volunteers (*n* = 72) were randomized to receive either placebo or one of two dose levels of fingolimod prior to immunization with 100 μg subunit KLH plus aluminium hydroxide adjuvant, once per week for three doses, followed by a 10 μg intradermal KLH dose to elicit a DTH response. Notably, fewer than two participants in each of the placebo and intervention arms (*n* = 24 each) developed a positive reaction (defined in this study as induration diameter >5 mm)—precluding the possibility of detecting an effect of the study drug. In the second, the effects of alternate immunosuppressive regimens used in patients who underwent renal transplantation on DTH, proliferative responses of peripheral blood mononuclear cell (PBMC) to KLH ex vivo, and immunohistochemical staining of punch skin biopsy at the site of intradermal KLH re‐challenge were investigated. “Vigorous” DTH responses were observed in healthy volunteers (*n* = 10) receiving 100 μg intradermal skin challenges with HMW‐KLH, 14 days following a primary intramuscular dose of 1000 μg of HMW‐KLH, in contrast to none of six patients who underwent renal transplantation treated for rejection with an anti‐CD3 mAb, and two of seven patients receiving standard immunosuppressive regimens (prednisolone and cyclosporin A ± mycophenolate mofetil).^51^ Conversely, there appeared to be limited differences between healthy volunteers and patients in terms of PBMC proliferative responses to ex vivo KLH stimulation. Immunohistochemistry identified a reduction of ICAM‐1 in the setting of immunosuppression with prednisolone/cyclosporin A, whereas E‐selectin, VCAM‐1, and CD3 expression were not affected.

### KLH challenge in other contexts

Twenty‐six of the 46 studies (57%) described application of KLH challenge in other contexts, including the effects of non‐pharmacological exposures or interventions (e.g., age, ultraviolet exposure, exercise, and psychosocial stress),^54^, ^55^, ^56^, ^57^, ^58^, ^59^, ^60^, ^61^ and the effect of disease states^62^, ^63^, ^64^, ^65^, ^66^, ^67^ on KLH response (Table S4). Two studies reported novel assays for assessing KLH‐specific immune responses.^14^, ^68^ The majority (21/26, 81%) of the studies in this category were published prior to 2014.

Selected studies identified in this category are relevant for development of the KLH challenge paradigm in general. Saghari et al.^29^ sought to characterize the performance characteristics of a single dose level KLH challenge protocol, with a particular focus on objective imaging techniques to quantify the DTH response. In this study, healthy male volunteers were administered subunit KLH (Biosyn, Carlsbad, CA) 100 μg (*n* = 12) with aluminium hydroxide adjuvant or placebo (*n* = 3) via intramuscular injection, followed by 1 μg intradermal injection 21 days later. The DTH response was assessed at 48 h, using clinical assessment (induration, erythema, tenderness, and pain), and by a variety of imaging modalities, including multispectral imaging (erythema and edema), colorimetry (erythema), and automated 2D photography (erythema). This study was notable for the low dose of subunit KLH used to elicit the DTH response, and associated excellent tolerance of the regimen. The DTH response was not apparent by visual inspection but could be identified using skin imaging. This regimen was subsequently applied to a phase I trial of an OX40L antagonist wherein modulation of the cutaneous DTH response by the IMP was demonstrated.^38^

Belson et al. characterized the DTH response in healthy volunteers receiving repeated skin challenges with HMW‐KLH or tuberculin/purified protein derivative (PPD), with a view to apply the paradigm to clinical trials of T‐cell targeting IMP.^28^ The KLH dose regimen was based on an earlier study reported as a conference abstract by Dickson et al.^27^ Healthy volunteers (*n* = 49) received various challenge regimens with KLH, PPD, or phosphate buffered saline. The DTH response was assessed clinically via measurement of erythema, and induration using the “ball‐point pen technique.”^39^ Laser Doppler imaging was used to measure erythema area and intensity. The DTH response was assessed at two timepoints (48 and 120 h post‐challenge). Intradermal KLH challenge and measurement of DTH reactions was performed on two occasions per participant (allowing estimation of interoccasion variability of the DTH response). In addition, the DTH reaction was assessed using both punch skin biopsy (immunohistochemistry) and analysis of suction blister fluid (cytokine concentrations and flow cytometry). The dose of HMW‐KLH used for subcutaneous primary dosing and intradermal re‐challenge was the highest identified (5000 μg and 100 μg, respectively), and consequently adverse effects were common: approximately half of the participants (11 of 21, 52%) reported an injection site reaction at 6–7 days following the primary dose. Based on better tolerability of the PPD regimen, for example, the KLH regimen (at the doses and formulation used), the authors concluded that the PPD regimen may be more appropriate for healthy volunteer studies. The PPD regimen was subsequently applied in a phase I/Ib study of an experimental LAG‐3 inhibitor developed by GlaxoSmithKline (GSK).^69^

Oyelaran et al. investigated antibody responses using a 107 component carbohydrate antigen microarray assay utilizing samples from an earlier study of healthy men (*n* = 14, younger and older cohorts) receiving 100 μg subunit KLH and aluminium hydroxide (900 μg).^14^, ^57^ Results from this study supported the hypothesis that the anti‐KLH antibody response in participants was largely directed at carbohydrate epitopes (rather than peptides), with considerable interindividual variability in the magnitude and breadth of responses to specific antigens, even among those with similar total anti‐KLH IgG response. In addition, the investigators identified a subset (13 of 107) of pre‐immune antibody responses, which were statistically significantly *inversely* correlated with the magnitude of the post‐immune anti‐KLH IgG response (at a *p* = 0.05 level without adjustment for multiple hypothesis testing), suggesting one potential method for accounting for some of the (marked) interindividual variability in anti‐KLH antibody responses as a function of baseline covariates.

### Use of adjuvants (all study types)

Ten of the 46 studies identified (22%) administered KLH with an adjuvant, namely aluminium hydroxide (7 studies), Montanide ISA‐51 (2 studies), and poly‐ICLC ± fimaporfin (1 study). In all but one study, these adjuvants were administered with subunit KLH (with the formulation not specified in the remaining study).

One study compared immune responses with and without adjuvants. Miller et al. described primary antibody and cellular responses (proliferation assays and anti‐IFNγ ELISpot) to KLH in healthy volunteers (*n* = 37) and a heterogenous group of patients with malignancies following chemotherapy (*n* = 14) or hemopoietic stem cell transplant (*n* = 19).^70^ In this study, participants were administered three different KLH containing regimens s.c.: (i) HMW‐KLH 1000 μg (Intracel, Rockville, MD), (ii) subunit KLH 1000 μg, or (iii) subunit KLH 1000 μg with Montanide ISA‐51 (Seppic, Fairfield, NJ). The allocation of challenge regimen was non‐randomized, and instead occurred due to unreliable supply of the HMW‐KLH product initially used, necessitating a change in supplier. HMW‐KLH induced a potent antibody response in healthy volunteers (*n* = 17), whereas healthy volunteers (*n* = 10) receiving the subunit product failed to mount a significant antibody or cellular response. Emulsification of the sub‐unit KLH with Montanide ISA‐51 adjuvant safely elicited a 100% antibody response rate in healthy volunteers (*n* = 10). Patients (*n* = 34), who received either HMW‐KLH or sub‐unit KLH with Montanide, demonstrated impaired responses (most pronounced in patients post‐hematopoietic stem cell transplant). The comparative effect of KLH formulation and Montanide on qualitative immune responses (e.g., T_H_1:T_H_2 skew) was not directly evaluated.

We identified one study reporting the application of a novel adjuvant strategy for promoting CD8+ T cell responses, via application of photochemical internalization (PCI)—a technique to induce the release of antigen from intracellular vesicles into the cytosol of antigen presenting cells in response to cutaneous laser illumination.^71^ In a phase I dose‐finding study, participants received two fortnightly intradermal doses of 100 μg subunit KLH (and human papillomavirus peptides) combined with a fixed dose (50 μg) of the TLR3 agonist poly‐ICLC (Hiltonol; Oncovir, Washington, DC) and ascending doses of the PCI agent fimaporfin. A control group received antigen and poly‐ICLC alone. All participants developed anti‐KLH IgG responses, with higher titers observed in participants receiving PCI with fimaporfin 12.5 μg compared to those in the control group. Fifty percent of participants receiving subunit KLH with poly‐ICLC alone developed positive IFN‐γ ELISpot responses, compared to 40%–100% of participants (depending on dose level) in groups receiving fimaporfin PCI plus poly‐ICLC where there was no clear relationship observed between fimaporfin dose and ELISpot response.

## DISCUSSION

### Principal findings

In this systematic review, we identified a per annum increase in studies using KLH‐HIC to assess the activity of immunomodulatory IMP in early phase clinical trials, suggesting greater interest in this approach over time. Whereas KLH challenge regimens were predominantly used to evaluate IMP targeting T‐cell co‐stimulatory pathways (immunosuppressive and immunostimulant), the paradigm was applied to the study of drugs exhibiting diverse immunomodulatory mechanisms. Most studies of immunomodulatory IMPs sought to demonstrate modulation of the anti‐KLH IgG response as a primary KLH‐specific outcome.

### Deficiencies in existing applications of KLH challenge

In most cases, the rationale for a particular aspect of KLH challenge study design was not described or was based on precedent set by a previous study. We observed marked and unjustified variability in fundamental aspects of study design. For example, primary KLH challenge doses varied 50‐fold (100–5000 μg, with a maximum dose of 2500 μg in early phase studies), whereas KLH doses used for elicitation of DTH response ranged 100‐fold between 1 and 100 μg. We identified no studies in the review period which explicitly sought to characterize KLH‐dose response, or to define an “optimal” dose. The consequence of poorly optimized challenge protocols is exemplified by studies in which the challenge regimen failed to elicit a response in an acceptable proportion of participants in any arm (including placebo), precluding an evaluation of the IMP using this end point,^35^, ^36^, ^50^ and studies observing high rates of injection site reactions to KLH.^28^

Heterogeneity was also observed with other aspects of design, including site and route of KLH administration, use of adjuvants, timing of KLH challenge relative to IMP administration, and both the nature and timing of outcome assessments. For example, of the two studies of immunosuppressive IMPs targeting the CD40‐CD40L interaction, one introduced KLH 14 days before the first IMP (dazodalibep) dose, whereas the other introduced KLH 3 days after the first IMP (iscalimab) dose.^43^, ^47^ The timing of KLH challenge relative to IMP would be expected to present distinct pathways elicited for potential modulation, although this was not explored. Overall, the available data suggest improved immunogenicity when subunit KLH is co‐administered with an adjuvant, although we identified no randomized controlled studies which evaluated this question. Although differential qualitative effects on the resultant immune response may be expected by varying the choice of adjuvant, this has yet to be systematically explored, and variability in participant characteristics, KLH challenge regimens, and methods of outcome assessment limits the conclusions which can be drawn by comparing responses across existing studies.

In some cases, the circumstances of a particular study will have driven rational decisions regarding KLH challenge regimen (e.g., high KLH doses used in the study of X‐linked hyper IgM patients),^34^ however, in most cases, variability in design was at best unexplained and at worst, arbitrary. Standardization of protocols (where appropriate) is likely to improve the generalizability and external validity of findings and, in turn, necessitates further research that systematically explores the operating characteristics of KLH challenge in order to develop a protocol that is fit‐for‐purpose for, or can be tailored to, a broad range of applications. A well‐characterized paradigm with established performance characteristics would inform sample size calculation and allow leverage of prior information (e.g., in Bayesian study designs). The value of this approach is likely to be high, especially in the setting of early phase trials of immunomodulatory agents that typically recruit small numbers of participants and which measure immunological endpoints, which are classically characterized by large interindividual variability—a combination of design characteristics which risks indeterminant results, erroneous inferences, and flawed development decisions. Standardization of procedures would additionally facilitate benchmarking between different IMPs targeting either the same or alternate mechanisms, enhancing insights into fundamental human immunology afforded by controlled perturbation. These considerations align with those seen in preclinical KLH challenge studies, including those performed in nonhuman primates and rats, where the effects of key covariates (e.g., study site, gender, KLH formulation, and use of adjuvants) has been quantified and used to inform power and sample size calculations.^17^, ^18^

Importantly, studies frequently failed to report key aspects of KLH challenge regimen, including formulation and dose of KLH. The absence of this (basic) information severely limits the reproducibility of study findings, and echoes ubiquitous reporting deficiencies identified throughout the medical literature.^72^, ^73^ Established guidelines exist for writing protocols and reporting of randomized controlled trials (SPIRIT^74^ and CONSORT,^75^ respectively, with extensions for early phase dose‐finding trials in progress^76^) and for the reporting of study interventions (TIDieR).^26^ There is, however, no guideline specific to the reporting of experimental medicine approaches, such as HIC, and none that we could identify as fit‐for‐purpose (without modification) for assessment of the studies in this review. As we have demonstrated, HIC can be performed as standalone research in healthy volunteer or patient populations, or integrated into studies with broader objectives (e.g., phase I clinical trials of IMPs). A reporting guideline specific to HIC (standalone or integrated into other studies, such as clinical trials), with sufficient detail to provide clear direction to authors, reviewers, and editors, may therefore be of great benefit for improving research reproducibility.

### From immunotoxicity assessment to proof of mechanism

A key observation of this review is the evolving application of KLH HIC from immunotoxicity assessment (mirroring its role in preclinical TDAR‐DTH studies^18^) to the assessment of PoM of immunomodulatory IMPs. In their systematic review of KLH HIC studies published between 1994 and 2013, Swaminathan et al. described applications which demonstrate the role of KLH as a probe for gauging the general immunosuppressive effect (manifesting as anti‐KLH IgG suppression) of various natural exposures, disease states, and assigned interventions (both pharmacological and non‐pharmacological), but notably, identified no early phase clinical studies.^13^

The increasing interest in use of KLH challenge to answer specific mechanistic questions highlights the limitations of study protocols as currently enacted. The choice of primary (KLH‐specific) outcome exemplifies this: anti‐KLH IgG is a useful and easily measured end point in immunotoxicity assessment, where the coordination of multiple processes of the innate and adaptive immune responses is required for successful IgG synthesis. Here, anti‐KLH IgG may be considered a sensitive marker of immunotoxicity, in a context where specific mechanisms of inhibition of antibody synthesis are of less interest. In contrast, when applied to immunomodulatory IMPs, or indeed to elucidation of basic human immunology, a higher resolution picture afforded by modern immunological techniques is arguably required. The inconsistent link between anti‐KLH IgG response and other PD markers discussed above illustrates the need to identify and characterize more mechanistically relevant KLH challenge endpoints which are fit‐for‐purpose for the PoM assessment of specific immunomodulatory IMPs. To provide just one example, whereas the study of Sullivan et al. asserted that evidence of anti‐KLH IgG modulation by AMG 557 (prezalumab) “demonstrates a PD effect of AMG 557 in subjects with SLE consistent with the biology of the ICOS pathway and supports further studies of AMG 557 as a potential therapeutic for autoimmune diseases,” this drug failed to meet primary end points in a subsequent phase II trial in SLE, and development has since been discontinued.

PoM assessment has been demonstrated to bridge an important translational gap between preclinical studies and clinical trials for selected disease indications, thereby improving the probability of success. The principles of PoM therefore may be an indispensable paradigm for decision making in early phase drug development. Successful application of PoM principles, however, requires careful attention to mechanism relevant immune pathways to demonstrate as clearly as possible whether the IMP under investigation is behaving in vivo as intended. A more ambitious goal would be to identify and validate pathways upregulated by HIC, which map to pathophysiological processes upregulated in discrete autoimmune/autoinflammatory conditions, but this is a remote prospect in the absence of careful fundamental characterization of KLH challenge's operating characteristics.

The KLH challenge paradigm is likely to be significantly strengthened through the use of modern immunological techniques on tissues relevant to the biophase of the IMP of interest (e.g., skin biopsy, skin blister fluid, and lymph node aspirates, collected at relevant timepoints; e.g., primary response, DTH response).^12^, ^77^ There is also an opportunity to explore the effects of adjuvants on the polarization of immune response to KLH, and to evaluate whether varying adjuvants can upregulate pathways relevant to a broader range of pharmacological mechanisms. These approaches and outcome assessments lend themselves to a quantitative systems pharmacology approach, which allows the development and evaluation of hypotheses relevant to the IMP of interest, and to more explicitly define the mechanisms which are being assessed in, for example, the clinical measurement of erythema or induration in a DTH response. Such approaches would also be of value in the qualitative and quantitative translation of preclinical KLH challenge studies to early phase clinical studies using the same IMP (as demonstrated in a limited number of studies identified in this review), and may improve understanding of the translational relevance of KLH challenge performed in different nonhuman species.^38^, ^42^ Table 3 summarizes key outstanding questions and potential opportunities for KLH HIC that may be addressed in future studies. Based on the results of this review, it may be premature to make specific recommendations for standardization of experimental protocols, prior to further work on the fundamental characterization and optimization of the paradigm.

**TABLE 3:** Outstanding questions for human immune challenge studies using KLH

Question	Potential value
*Design of challenge regimen*	
What is the optimal dose (primary and intradermal re‐challenge) of KLH for HIC?	Standardization of dosing between studies to improve benchmarking and generalizability of findings
How does response to intradermal KLH following initial immunization vary over time, and upon multiple re‐exposures?	Establish the potential of within‐subject‐designs for PoM assessment and determine the sensitivity of responses to timing of rechallenge
How might different adjuvants influence immune response polarization to KLH, and can this be used to activate mechanism‐ and disease‐relevant immune pathways for further interrogation?	Controlled elicitation of specific immune responses (e.g., T_H_1 vs. T_H_2 vs. T_H_17) may improve the relevance and applicability of the model to mechanistic assessment of a larger range of immunomodulatory IMPs
*Outcome assessment*	
Can assessment of KLH‐specific immune response in relevant tissues (e.g., skin biopsy, skin blister fluid, lymph node aspirate) provide enhanced insight over peripheral blood samples?	Assessment of modulation of stromal immune responses by IMP may provide more mechanism‐relevant information regarding IMP activity
What are the determinants of the nature and magnitude of the immune response to KLH, can it be predicted, and how does it vary (qualitatively and quantitatively) between individuals?	Provide prior information to inform study design (e.g., sample size calculations, and prior distributions for Bayesian statistical approaches)
What is the time course of the blood and stromal response to primary KLH immunization and rechallenge?	Identify timepoints for outcome assessment relevant to a mechanism of interest for a given IMP
*Maximizing learning from HIC studies employing KLH*	
Can systems pharmacology and immunology approaches be used to leverage existing knowledge and elucidate response to KLH and its modulation by IMP?	Early phase clinical trials typically recruit small numbers of participants. Leveraging existing mechanistic knowledge may ameliorate the limited power or uncertain inferences based on simple frequentist approaches to statistical analyses of study outcome data.

Abbreviations: HIC, human immune challenge; IMPs, investigational medicinal products; KLH, keyhole limpet hemocyanin; PoM, proof of mechanism.

### Strengths and limitations

In this study, we present a comprehensive survey of the KLH HIC literature, and our focus on applications to drug development represents a distinct contribution when compared to earlier reviews.^13^, ^15^ We used a comprehensive strategy and standardized approach to extract relevant study features, although there are some limitations. Given that KLH challenge is not usually the primary focus of a published study, its use is frequently not highlighted in study titles, abstracts, or keywords. We have attempted to identify all relevant studies using a comprehensive search strategy, including cross‐referencing with clinical trials registries and searching of reference lists, although it is possible some relevant studies have not been identified, especially where KLH challenge is not specifically referenced in the title or abstract of the study.

We chose to focus on the application of KLH HIC to drug development, and as such our analysis of KLH challenge applications in other settings (e.g., effects of non‐pharmacological exposures, physiological states, and diseases states) was more limited. As demonstrated in Figure 2, the incidence of these studies has reduced since 2014, and as such the previous review by Swaminathan et al.^13^ gives a suitably comprehensive account of these studies. The deficiencies in KLH challenge identified in our review are likely to have similar implications on the validity, reproducibility, and immunological insights afforded through these applications of the paradigm.

Finally, this review focused on HIC utilizing KLH only. Numerous antigens can be used to elicit a T‐cell dependent immune response in humans, including tuberculin/purified protein derivative, tetanus, varicella, candida, and others—a full survey of candidate antigens for HIC was beyond the scope of this review, and it is possible that an alternative antigen may be preferred for HIC in certain contexts. KLH has several attributes which favor its use for HIC, including xenogenic origin, such that most volunteers are immunologically naïve to relevant epitopes (although a small proportion of KLH‐unexposed people will have cross‐reactive immune responses), longstanding use (including in the preclinical setting), excellent safety record, and availability of a GMP‐grade product. The value of KLH HIC should be evaluated in the context of complementary information that may be elicited by other pharmacological assays (e.g., target occupancy assays), which may be deployed in the evaluation of IMPs in early phase clinical trials. Ultimately, PoM determination should be based on a total weight of high‐quality evidence assessment.

## CONCLUSION

KLH has an established role in immunotoxicology assessment with demonstrated predictive value in multiple nonhuman species. The studies identified in this systematic review attest to the value of human KLH challenge as a platform for interrogating a broad range of pharmacological mechanisms, translating preclinical data, and ultimately informing decision making in early phase clinical trials. They also highlight significant methodological and technical heterogeneity, and a historical reliance on simplistic, mechanism‐independent biomarkers as PD end points. Explicit determination of the operating characteristics of the model, expansion of the range of immunological pathways elicited through alternate immunization protocols, acquisition of relevant biological samples and application of modern immunological techniques to KLH‐driven responses in multiple tissue compartments, and clear comprehensive reporting of the results of these studies will maximize the latent potential of the paradigm.

## FUNDING INFORMATION

This work was supported by the Medical Research Council (UK) (MR/S025308/1) and the Kennedy Trust for Rheumatology Research (KENN 20 21 02).

## CONFLICT OF INTEREST

The authors declared no competing interests for this work.

## Supporting information


Tables S1‐S5.

## Footnotes

^b^
The phase 1b MAD study reported in the same publication recruited patients with rheumatoid arthritis (*n* = 57). These participants did not receive KLH challenge.
