# Supplementary material for: In vivo human keyhole limpet hemocyanin challenge in early phase drug development: A systematic review
Source: Clin Transl Sci. 2022 Nov 24;16(3):357–82. doi: 10.1111/cts.13457 (PMC10014697; doi:10.1111/cts.13457)
Supplement: Supplementary file 1 — Tables S1‐S5. [file CTS-16-357-s001.docx]

**Supplementary material for: *In vivo* human keyhole limpet haemocyanin challenge in early phase drug development: A systematic review**

**Table S1: PRISMA Checklist**

|  |  | Reporting Item | Page Number |
| --- | --- | --- | --- |
| **Title** |  |  |  |
| Title | [#1](https://www.goodreports.org/reporting-checklists/prisma/info/#1) | Identify the report as a systematic review | 1 |
| **Abstract** |  |  |  |
| Abstract | [#2](https://www.goodreports.org/reporting-checklists/prisma/info/#2) | Report an abstract addressing each item in the PRISMA 2020 for Abstracts checklist | 2 |
| **Introduction** |  |  |  |
| Background/rationale | [#3](https://www.goodreports.org/reporting-checklists/prisma/info/#3) | Describe the rationale for the review in the context of existing knowledge | 3 |
| Objectives | [#4](https://www.goodreports.org/reporting-checklists/prisma/info/#4) | Provide an explicit statement of the objective(s) or question(s) the review addresses | 7 |
| **Methods** |  |  |  |
| Eligibility criteria | [#5](https://www.goodreports.org/reporting-checklists/prisma/info/#5) | Specify the inclusion and exclusion criteria for the review and how studies were grouped for the syntheses | 7-8 |
| Information sources | [#6](https://www.goodreports.org/reporting-checklists/prisma/info/#6) | Specify all databases, registers, websites, organisations, reference lists, and other sources searched or consulted to identify studies. Specify the date when each source was last searched or consulted | 7 |
| Search strategy | [#7](https://www.goodreports.org/reporting-checklists/prisma/info/#7) | Present the full search strategies for all databases, registers, and websites, including any filters and limits used | s2 |
| Selection process | [#8](https://www.goodreports.org/reporting-checklists/prisma/info/#8) | Specify the methods used to decide whether a study met the inclusion criteria of the review, including how many reviewers screened each record and each report retrieved, whether they worked independently, and, if applicable, details of automation tools used in the process | 7 |
| Data collection process | [#9](https://www.goodreports.org/reporting-checklists/prisma/info/#9) | Specify the methods used to collect data from reports, including how many reviewers collected data from each report, whether they worked independently, any processes for obtaining or confirming data from study investigators, and, if applicable, details of automation tools used in the process | 7 |
| Data items | [#10a](https://www.goodreports.org/reporting-checklists/prisma/info/#10a) | List and define all outcomes for which data were sought. Specify whether all results that were compatible with each outcome domain in each study were sought (for example, for all measures, time points, analyses), and, if not, the methods used to decide which results to collect | 7 |
| Study risk of bias assessment | [#11](https://www.goodreports.org/reporting-checklists/prisma/info/#11) | Specify the methods used to assess risk of bias in the included studies, including details of the tool(s) used, how many reviewers assessed each study and whether they worked independently, and, if applicable, details of automation tools used in the process | No RoB assessment, reporting quality assessment page 8 |
| Effect measures | [#12](https://www.goodreports.org/reporting-checklists/prisma/info/#12) | Specify for each outcome the effect measure(s) (such as risk ratio, mean difference) used in the synthesis or presentation of results | NA |
| Synthesis methods | [#13a](https://www.goodreports.org/reporting-checklists/prisma/info/#13a) | Describe the processes used to decide which studies were eligible for each synthesis (such as tabulating the study intervention characteristics and comparing against the planned groups for each synthesis (item #5)) | NA |
| Synthesis methods | [#13b](https://www.goodreports.org/reporting-checklists/prisma/info/#13b) | Describe any methods required to prepare the data for presentation or synthesis, such as handling of missing summary statistics or data conversions | NA |
| Synthesis methods | [#13c](https://www.goodreports.org/reporting-checklists/prisma/info/#13c) | Describe any methods used to tabulate or visually display results of individual studies and syntheses | NA |
| Synthesis methods | [#13d](https://www.goodreports.org/reporting-checklists/prisma/info/#13d) | Describe any methods used to synthesise results and provide a rationale for the choice(s). If meta-analysis was performed, describe the model(s), method(s) to identify the presence and extent of statistical heterogeneity, and software package(s) used | NA |
| Synthesis methods | [#13e](https://www.goodreports.org/reporting-checklists/prisma/info/#13e) | Describe any methods used to explore possible causes of heterogeneity among study results (such as subgroup analysis, meta-regression) | NA |
| Synthesis methods | [#13f](https://www.goodreports.org/reporting-checklists/prisma/info/#13f) | Describe any sensitivity analyses conducted to assess robustness of the synthesised results | NA |
| Reporting bias assessment | [#14](https://www.goodreports.org/reporting-checklists/prisma/info/#14) | Describe any methods used to assess risk of bias due to missing results in a synthesis (arising from reporting biases) | NA |
| Certainty assessment | [#15](https://www.goodreports.org/reporting-checklists/prisma/info/#15) | Describe any methods used to assess certainty (or confidence) in the body of evidence for an outcome | NA |
| Data items | [#10b](https://www.goodreports.org/reporting-checklists/prisma/info/#10b) | List and define all other variables for which data were sought (such as participant and intervention characteristics, funding sources). Describe any assumptions made about any missing or unclear information | 7 |
| **Results** |  |  |  |
| Study selection | [#16a](https://www.goodreports.org/reporting-checklists/prisma/info/#16a) | Describe the results of the search and selection process, from the number of records identified in the search to the number of studies included in the review, ideally using a flow diagram (http://www.prisma-statement.org/PRISMAStatement/FlowDiagram) | Fig1 |
| Study selection | [#16b](https://www.goodreports.org/reporting-checklists/prisma/info/#16b) | Cite studies that might appear to meet the inclusion criteria, but which were excluded, and explain why they were excluded | 14 |
| Study characteristics | [#17](https://www.goodreports.org/reporting-checklists/prisma/info/#17) | Cite each included study and present its characteristics | 9 |
| Risk of bias in studies | [#18](https://www.goodreports.org/reporting-checklists/prisma/info/#18) | Present assessments of risk of bias for each included study | NA |
| Results of individual studies | [#19](https://www.goodreports.org/reporting-checklists/prisma/info/#19) | For all outcomes, present for each study (a) summary statistics for each group (where appropriate) and (b) an effect estimate and its precision (such as confidence/credible interval), ideally using structured tables or plots | 9 |
| Results of syntheses | [#20a](https://www.goodreports.org/reporting-checklists/prisma/info/#20a) | For each synthesis, briefly summarise the characteristics and risk of bias among contributing studies | NA |
| Results of syntheses | [#20b](https://www.goodreports.org/reporting-checklists/prisma/info/#20b) | Present results of all statistical syntheses conducted. If meta-analysis was done, present for each the summary estimate and its precision (such as confidence/credible interval) and measures of statistical heterogeneity. If comparing groups, describe the direction of the effect | NA |
| Results of syntheses | [#20c](https://www.goodreports.org/reporting-checklists/prisma/info/#20c) | Present results of all investigations of possible causes of heterogeneity among study results | NA |
| Results of syntheses | [#20d](https://www.goodreports.org/reporting-checklists/prisma/info/#20d) | Present results of all sensitivity analyses conducted to assess the robustness of the synthesised results | NA |
| Risk of reporting biases in syntheses | [#21](https://www.goodreports.org/reporting-checklists/prisma/info/#21) | Present assessments of risk of bias due to missing results (arising from reporting biases) for each synthesis assessed | NA |
| Certainty of evidence | [#22](https://www.goodreports.org/reporting-checklists/prisma/info/#22) | Present assessments of certainty (or confidence) in the body of evidence for each outcome assessed | NA |
| **Discussion** |  |  |  |
| Results in context | [#23a](https://www.goodreports.org/reporting-checklists/prisma/info/#23a) | Provide a general interpretation of the results in the context of other evidence | 17 |
| Limitations of included studies | [#23b](https://www.goodreports.org/reporting-checklists/prisma/info/#23b) | Discuss any limitations of the evidence included in the review | 20 |
| Limitations of the review methods | [#23c](https://www.goodreports.org/reporting-checklists/prisma/info/#23c) | Discuss any limitations of the review processes used | 20 |
| Implications | [#23d](https://www.goodreports.org/reporting-checklists/prisma/info/#23d) | Discuss implications of the results for practice, policy, and future research | 20 |
| **Other information** |  |  |  |
| Registration and protocol | [#24a](https://www.goodreports.org/reporting-checklists/prisma/info/#24a) | Provide registration information for the review, including register name and registration number, or state that the review was not registered | n/a |
| Registration and protocol | [#24b](https://www.goodreports.org/reporting-checklists/prisma/info/#24b) | Indicate where the review protocol can be accessed, or state that a protocol was not prepared | NA |
| Registration and protocol | [#24c](https://www.goodreports.org/reporting-checklists/prisma/info/#24c) | Describe and explain any amendments to information provided at registration or in the protocol | NA |
| Support | [#25](https://www.goodreports.org/reporting-checklists/prisma/info/#25) | Describe sources of financial or non-financial support for the review, and the role of the funders or sponsors in the review | 1 |
| Competing interests | [#26](https://www.goodreports.org/reporting-checklists/prisma/info/#26) | Declare any competing interests of review authors | 1 |
| Availability of data, code, and other materials | [#27](https://www.goodreports.org/reporting-checklists/prisma/info/#27) | Report which of the following are publicly available and where they can be found: template data collection forms; data extracted from included studies; data used for all analyses; analytic code; any other materials used in the review | 1 |

**Table S2: Search Strategy**

| Clinicaltrials.gov  KLH OR "keyhole limpet hemocyanin" = 150 trials  Records selected for further review = 25 |
| --- |
| Cochrane Central search date 30 April 2022  “KLH” or “Keyhole Limpet” = 258 trials  Records selected for further review = 44 |
| OVID Medline search date 30 April 2022  Medline (Ovid MEDLINE® Epub Ahead of Print, In-Process & Other Non-Indexed Citations, Ovid MEDLINE® Daily and Ovid MEDLINE®) 1946 to present  1 KLH.mp. 2970  2 keyhole limpet hemocyanin.mp. 3871  3 keyhole limpet haemocyanin.mp. 504  4 1 or 2 or 3 4918  5 exp animals/ not humans.sh. 5001776  6 mice.mp. or Mice/ 1847098  7 bladder cancer.mp. or Urinary Bladder Neoplasms/ 69969  8 5 or 6 or 7 5789490  9 4 not 8 1145  10 limit 9 to yr="1994 -Current" 847  11 from 10 keep 16,23,40-41,44,50,56,58,63,84,103,109,138,161-162,170,189,223,226,274,292,298,303,326,353,432,478,481,486,491,502,520,558,566,660,664,675,679,694,751,755,776,780,816  Records selected for further review = 44 |
| OVID EMBASE search date 30 April 2022  Embase 1974 to present  1 keyhole limpet hemocyanin/ 3365  2 KLH.mp. 3743  3 1 or 2 5402  4 exp human/ or human tissue.mp. [mp=title, abstract, heading word, drug trade name, original title, device manufacturer, drug manufacturer, device trade name, keyword heading word, floating subheading word, candidate term word] 23574384  5 exp animal/ or exp invertebrate/ or nonhuman/ or animal experiment/ or animal tissue/ or animal model/ or exp plant/ or exp fungus/ 30903717  6 5 not 4 7343949  7 3 not 6 2229  8 cancer vaccine/ or tumor vaccine/ or lymphoma vaccine/ or dendritic cell vaccine/ 23618  9 bladder cancer/ 54414  10 8 or 9 77630  11 7 not 10 1644  12 limit 11 to yr="1994 -Current" 1344  13 in vitro study/ 1411012  14 12 not 13 1220  15 malignant neoplasm/ 77761  16 14 not 15 1216  17 lymphoma/ 110156  18 16 not 17 1197  19 from 19 keep 2,21-22,34,41,44,60,70-71,87-88,97,99,119,122,127,131,137,142,155-156,190,197,210-211,219,227,235,237,241,249,291-292,305,331,352,371-372,399,404,424,514,579,589,595,647,689,792,809,861,876,878,912,946,997,1083-1084,1139  Records selected for further review = 58 |

**Table S3: Modified TIDieR reporting checklist for early phase clinical trials of IMP employing KLH challenge. Numbers refer to sections of original TIDieR checklist.**

| Checklist component | Guidance notes |
| --- | --- |
| 2a. Describes rationale for using KLH challenge | Minimum requirement is some specificity. E.g. 'to examine PD effect' would be insufficient, but 'to examine effects on neoantigen response' would be sufficient. |
| 2a: detail | what was the rationale for using KLH challenge |
| 2b. Describes rationale for a specific challenge regimen | Score yes if *any* discussion of rationale (e.g. why specific endpoint or timings chosen) |
| 2b: detail | What was the rationale for the specific challenge regimen |
| 3a. Describes KLH form |  |
| 3b. Describes KLH product name |  |
| 3c. Describes KLH supplier/company |  |
| 3d. Describes adjuvant generic name |  |
| Describes adjuvant product name |  |
| 3f. Describes adjuvant supplier |  |
| 4a. Describes KLH preparation procedure |  |
| 4b. Describes KLH route of administration |  |
| 4c. Describes KLH site of administration |  |
| 4d. Describes rechallenge route of administration |  |
| 4e. Describes rechallenge site of administration |  |
| 4f. Describes KLH administration device | e.g. ‘27g needle’ |
| 5a. Describes who administered the challenge agent | e.g. administered by trained study staff |
| 7. Describes where the study was conducted |  |
| 8a. Describes primary KLH dose |  |
| 8b. Describes primary KLH timepoint relative to other study interventions |  |
| 8c. Describes rechallenge KLH doses |  |
| 8d. Describes rechallenge KLH timepoints |  |
| 8e. Describes adjuvant dose |  |
| 9. Describes use of KLH challenge in title |  |
| 10a. Describes use of KLH challenge in abstract |  |
| 10b. Refers to assessment of immune response of an administered antigen in abstract |  |
| Other: Blinding: were assessors of KLH DTH clinical response blinded to dose level or placebo | NA if no placebo, single dose level, or if no DTH performed) |

**Table S4: Other human studies employing KLH challenge (n=26)**

| Lead author | Year Published | Title | Aim |
| --- | --- | --- | --- |
| Kondratenko^62^ | 1997 | Lack of specific antibody response in common variable immunodeficiency (CVID) associated with failure in production of antigen-specific memory T cells. MRC Immunodeficiency Group | To evaluate responses to primary KLH immunization in patients with immunodeficiency states |
| Kuijpers^87^ | 1997 | Antigen-specific immune responsiveness and lymphocyte recruitment in leukocyte adhesion deficiency type II | To explore the in vivo immune responsiveness and lymphocyte recruitment to the skin, in response to the neo-antigen keyhole limpet hemocyanin (KLH) in a LAD-II patient |
| Suchin^88^ | 1999 | Extracorporeal photochemotherapy does not suppress T- or B-cell responses to novel or recall antigens | To determine the effect of photopheresis on humoral and cell-mediated immunity in human subjects |
| Valdez^67^ | 2000 | Response to immunization with recall and neoantigens after prolonged administration of an HIV-1 protease inhibitor-containing regimen. ACTG 375 team. AIDS Clinical Trials Group | To assess response to immunization after prolonged anti-retroviral therapy in patients with HIV |
| Markert^89^ | 2000 | Effect of highly active antiretroviral therapy and thymic transplantation on immunoreconstitution in HIV infection | To address whether thymus transplantation in concert with HAART would facilitate the regeneration of the immune system in HIV infection |
| Lange^63^ | 2003 | Nadir CD4+ T-cell count and numbers of CD28+ CD4+ T-cells predict functional responses to immunizations in chronic HIV-1 infection | To ascertain whether delaying the initiation of highly active antiretroviral therapy compromises functional immune constitution in HIV-1 infection in persons who regain 'normal' CD4 T-cell counts after suppressive antiretroviral therapies |
| Lange^64^ | 2003 | Proliferation responses to HIVp24 during antiretroviral therapy do not reflect improved immune phenotype or function | To ascertain whether lymphoproliferation (LP) responses to HIVp24 in chronically infected patients treated with ART predict and improved cytolytic T-cell phenotype or better in vivo immune function as measured by immunisation responses |
| Boelens^60^ | 2003 | Glutamine-enriched enteral nutrition increases in vitro interferon-gamma production but does not influence the in vivo specific antibody response to KLH after severe trauma. A prospective, double blind, randomized clinical study | To compare the effect of glutamine-enriched enteral nutrition with control enteral feeding on type I and type II T-lymphocyte responses in patients with severe trauma |
| Smith^57^ | 2004 | Influence of age and physical activity on the primary in vivo antibody and T cell-mediated responses in men | To examine the effect of age and physical activity on primary immune response to KLH immunization |
| Boelens^59^ | 2004 | Primary immune response to keyhole limpet haemocyanin following trauma in relation to low plasma glutamine | To examine the effect of severe trauma on early primary immune response to KLH immunization in relation to low plasma glutathione |
| Smith^56^ | 2004 | The relationship between distress and the development of a primary immune response to a novel antigen | To examine the effect of distress on primary KLH immunization response in young adults |
| Smith^58^ | 2004 | Influences of distress and alcohol consumption on the development of a delayed-type hypersensitivity skin test response | To examine the effect of psychological distress on DTH response following primary KLH immunization in young adults |
| Miller^70^ | 2005 | Diminished neo-antigen response to keyhole limpet hemocyanin (KLH) vaccines in patients after treatment with chemotherapy or hematopoietic cell transplantation | To compare the responses to KLH immunization in healthy adults with those in immunosuppressed patients (cancer and bone marrow transplant recipients) |
| Grant^55^ | 2008 | Cardiovascular exercise intervention improves the primary antibody response to keyhole limpet hemocyanin (KLH) in previously sedentary older adults | To examine the effect of aerobic exercise in sedentary older adults on primary immune response to KLH immunization |
| Spazierer^90^ | 2009 | T helper 2 biased de novo immune response to Keyhole Limpet Hemocyanin in humans | To establish an immunization protocol to induce de novo Th2 responses using immunization with KLH |
| Oyelaran^14^ | 2010 | Evaluation of human antibody responses to keyhole limpet hemocyanin on a carbohydrate microarray | To evaluate antibody responses to KLH in humans using a novel carbohydrate microarray |
| Rodriguez^65^ | 2010 | In vitro naive T cell proliferation failure predicts poor post-immunization responses to neoantigen, but not recall antigens, in HIV-infection | To investigate whether naive T cell proliferation could predict in vivo responses to immunization in HIV |
| Milgrom^91^ | 2012 | Response to cutaneous immunization with low-molecular-weight subunit keyhole limpet hemocyanin | To determine whether humoral and cellular immune responses would be provoked by cutaneous administration of keyhole limpet hemocyanin and in particular by scarification of the skin |
| Ferbas^68^ | 2013 | A novel assay to measure B cell responses to keyhole limpet haemocyanin vaccination in healthy volunteers and subjects with systemic lupus erythematosus | To assess performance characteristics of immunoassays measuring antigen specific response to KLH immunization in healthy controls and patients with systemic lupus erythematosus |
| Gallegos^54^ | 2013 | Toward identifying the effects of the specific components of Mindfulness-Based Stress Reduction on biologic and emotional outcomes among older adults | To examine the effects of mindfulness-based stress reduction on immunological outcomes in older adults |
| Moynihan^92^ | 2013 | Mindfulness-Based Stress Reduction for Older Adults: Effects on Executive Function, Frontal Alpha Asymmetry and Immune Function | To explore the effect of mindfulness based stress reduction for older adults on executive function, left frontal asymmetry and antibody response |
| Belson^28^ | 2016 | Characterisation of the clinical and activated T cell response to repeat delayed-type hypersensitivity skin challenges in human subjects, with KLH and PPD, as a potential model to test T cell-targeted therapies | To characterise the delayed-type hypersensitivity (DTH) skin reaction to repeated challenges of keyhole limpet hemocyanin (KLH) and tuberculin purified protein derivative (PPD) in healthy volunteers, as a potential model to test T cell-targeted investigational agents. |
| Giesecke^93^ | 2018 | Simultaneous Presence of Non- and Highly Mutated Keyhole Limpet Hemocyanin (KLH)-Specific Plasmablasts Early after Primary KLH Immunization Suggests Cross-Reactive Memory B Cell Activation | To describe the progression of human primary and secondary humoral immunity following KLH administration |
| Swaminathan^61^ | 2019 | Exposure to Solar UVR Suppresses Cell-Mediated Immunization Responses in Humans: The Australian Ultraviolet Radiation and Immunity Study | To assess the influence of natural sun exposure on the sensitization phase of a primary immune response to a model protein antigen, keyhole limpet hemocyanin |
| Saghari^29^ | 2020 | A randomized controlled trial with a delayed‐type hypersensitivity model using keyhole limpet haemocyanin to evaluate adaptive immune responses in man | To objectively quantify KLH-specific DTH responses, in relation to KLH-specific circulating antibody responses, using skin blood perfusion measurements |
| Otterhaug^71^ | 2021 | Photochemical Internalization Enhanced Vaccination Is Safe, and Gives Promising Cellular Immune Responses to an HPV Peptide-Based Vaccine in a Phase I Clinical Study in Healthy Volunteers | To assess the safety and local tolerance of phytochemical internalisation mediated vaccination |

| Lead author | Year Published | Title | Aim |
| --- | --- | --- | --- |
| Kondratenko^62^ | 1997 | Lack of specific antibody response in common variable immunodeficiency (CVID) associated with failure in production of antigen-specific memory T cells. MRC Immunodeficiency Group | To evaluate responses to primary KLH immunization in patients with immunodeficiency states |
| Kuijpers^87^ | 1997 | Antigen-specific immune responsiveness and lymphocyte recruitment in leukocyte adhesion deficiency type II | To explore the in vivo immune responsiveness and lymphocyte recruitment to the skin, in response to the neo-antigen keyhole limpet hemocyanin (KLH) in a LAD-II patient |
| Suchin^88^ | 1999 | Extracorporeal photochemotherapy does not suppress T- or B-cell responses to novel or recall antigens | To determine the effect of photopheresis on humoral and cell-mediated immunity in human subjects |
| Valdez^67^ | 2000 | Response to immunization with recall and neoantigens after prolonged administration of an HIV-1 protease inhibitor-containing regimen. ACTG 375 team. AIDS Clinical Trials Group | To assess response to immunization after prolonged anti-retroviral therapy in patients with HIV |
| Markert^89^ | 2000 | Effect of highly active antiretroviral therapy and thymic transplantation on immunoreconstitution in HIV infection | To address whether thymus transplantation in concert with HAART would facilitate the regeneration of the immune system in HIV infection |
| Lange^63^ | 2003 | Nadir CD4+ T-cell count and numbers of CD28+ CD4+ T-cells predict functional responses to immunizations in chronic HIV-1 infection | To ascertain whether delaying the initiation of highly active antiretroviral therapy compromises functional immune constitution in HIV-1 infection in persons who regain 'normal' CD4 T-cell counts after suppressive antiretroviral therapies |
| Lange^64^ | 2003 | Proliferation responses to HIVp24 during antiretroviral therapy do not reflect improved immune phenotype or function | To ascertain whether lymphoproliferation (LP) responses to HIVp24 in chronically infected patients treated with ART predict and improved cytolytic T-cell phenotype or better in vivo immune function as measured by immunisation responses |
| Boelens^60^ | 2003 | Glutamine-enriched enteral nutrition increases in vitro interferon-gamma production but does not influence the in vivo specific antibody response to KLH after severe trauma. A prospective, double blind, randomized clinical study | To compare the effect of glutamine-enriched enteral nutrition with control enteral feeding on type I and type II T-lymphocyte responses in patients with severe trauma |
| Smith^57^ | 2004 | Influence of age and physical activity on the primary in vivo antibody and T cell-mediated responses in men | To examine the effect of age and physical activity on primary immune response to KLH immunization |
| Boelens^59^ | 2004 | Primary immune response to keyhole limpet haemocyanin following trauma in relation to low plasma glutamine | To examine the effect of severe trauma on early primary immune response to KLH immunization in relation to low plasma glutathione |
| Smith^56^ | 2004 | The relationship between distress and the development of a primary immune response to a novel antigen | To examine the effect of distress on primary KLH immunization response in young adults |
| Smith^58^ | 2004 | Influences of distress and alcohol consumption on the development of a delayed-type hypersensitivity skin test response | To examine the effect of psychological distress on DTH response following primary KLH immunization in young adults |
| Miller^70^ | 2005 | Diminished neo-antigen response to keyhole limpet hemocyanin (KLH) vaccines in patients after treatment with chemotherapy or hematopoietic cell transplantation | To compare the responses to KLH immunization in healthy adults with those in immunosuppressed patients (cancer and bone marrow transplant recipients) |
| Grant^55^ | 2008 | Cardiovascular exercise intervention improves the primary antibody response to keyhole limpet hemocyanin (KLH) in previously sedentary older adults | To examine the effect of aerobic exercise in sedentary older adults on primary immune response to KLH immunization |
| Spazierer^90^ | 2009 | T helper 2 biased de novo immune response to Keyhole Limpet Hemocyanin in humans | To establish an immunization protocol to induce de novo Th2 responses using immunization with KLH |
| Oyelaran^14^ | 2010 | Evaluation of human antibody responses to keyhole limpet hemocyanin on a carbohydrate microarray | To evaluate antibody responses to KLH in humans using a novel carbohydrate microarray |
| Rodriguez^65^ | 2010 | In vitro naive T cell proliferation failure predicts poor post-immunization responses to neoantigen, but not recall antigens, in HIV-infection | To investigate whether naive T cell proliferation could predict in vivo responses to immunization in HIV |
| Milgrom^91^ | 2012 | Response to cutaneous immunization with low-molecular-weight subunit keyhole limpet hemocyanin | To determine whether humoral and cellular immune responses would be provoked by cutaneous administration of keyhole limpet hemocyanin and in particular by scarification of the skin |
| Ferbas^68^ | 2013 | A novel assay to measure B cell responses to keyhole limpet haemocyanin vaccination in healthy volunteers and subjects with systemic lupus erythematosus | To assess performance characteristics of immunoassays measuring antigen specific response to KLH immunization in healthy controls and patients with systemic lupus erythematosus |
| Gallegos^54^ | 2013 | Toward identifying the effects of the specific components of Mindfulness-Based Stress Reduction on biologic and emotional outcomes among older adults | To examine the effects of mindfulness-based stress reduction on immunological outcomes in older adults |
| Moynihan^92^ | 2013 | Mindfulness-Based Stress Reduction for Older Adults: Effects on Executive Function, Frontal Alpha Asymmetry and Immune Function | To explore the effect of mindfulness based stress reduction for older adults on executive function, left frontal asymmetry and antibody response |
| Belson^28^ | 2016 | Characterisation of the clinical and activated T cell response to repeat delayed-type hypersensitivity skin challenges in human subjects, with KLH and PPD, as a potential model to test T cell-targeted therapies | To characterise the delayed-type hypersensitivity (DTH) skin reaction to repeated challenges of keyhole limpet hemocyanin (KLH) and tuberculin purified protein derivative (PPD) in healthy volunteers, as a potential model to test T cell-targeted investigational agents. |
| Giesecke^93^ | 2018 | Simultaneous Presence of Non- and Highly Mutated Keyhole Limpet Hemocyanin (KLH)-Specific Plasmablasts Early after Primary KLH Immunization Suggests Cross-Reactive Memory B Cell Activation | To describe the progression of human primary and secondary humoral immunity following KLH administration |
| Swaminathan^61^ | 2019 | Exposure to Solar UVR Suppresses Cell-Mediated Immunization Responses in Humans: The Australian Ultraviolet Radiation and Immunity Study | To assess the influence of natural sun exposure on the sensitization phase of a primary immune response to a model protein antigen, keyhole limpet hemocyanin |
| Saghari^29^ | 2020 | A randomized controlled trial with a delayed‐type hypersensitivity model using keyhole limpet haemocyanin to evaluate adaptive immune responses in man | To objectively quantify KLH-specific DTH responses, in relation to KLH-specific circulating antibody responses, using skin blood perfusion measurements |
| Otterhaug^71^ | 2021 | Photochemical Internalization Enhanced Vaccination Is Safe, and Gives Promising Cellular Immune Responses to an HPV Peptide-Based Vaccine in a Phase I Clinical Study in Healthy Volunteers | To assess the safety and local tolerance of phytochemical internalisation mediated vaccination |

**Table S5: Consensus TIDieR checklist for identified early phase clinical trials utilising KLH challenge (n=14)**

| **lead_author** | **Abrams** | **Jain** | **Curti** | **Poirier** | **Shi** | **Sullivan** | **St Clair** |
| --- | --- | --- | --- | --- | --- | --- | --- |
| **title** | CTLA4Ig-mediated blockade of T-cell costimulation in patients with psoriasis vulgaris | Partial immune reconstitution of X-linked hyper IgM syndrome with recombinant CD40 ligand | OX40 is a potent immune stimulating target in late stage cancer patients | First-in-Human Study in Healthy Subjects with FR104, a Pegylated Monoclonal Antibody Fragment Antagonist of CD28 | Pharmacokinetic, Pharmacodynamic, and Safety Profile of a Novel Anti-CD28 Domain Antibody Antagonist in Healthy Subjects | Inducible T-cell co-stimulator ligand (ICOSL) blockade leads to selective inhibition of anti-KLH IgG responses in subjects with systemic lupus erythematosus | The Clinical Efficacy and Safety of Baminercept, a Lymphotoxin-β Receptor Fusion Protein, in Primary Sjögren’s Syndrome: Results from a Randomized, Double-Blind, Placebo-Controlled Phase II Trial |
| **2a. Describes rationale for using KLH challenge** | yes | yes | no | yes | yes | yes | yes |
| **2a detail (what was the rationale for using KLH challenge)** | To determine the ability of CTLA-4Ig to alter a humoral immune response to two T-dependent neoantigens. | To determine the effect of rCD40L on DTH responses to KLH | not directly described in methods | To gain access to preliminary efficacy in controlling an antibody response to a KLH antigen challenge | To establish range of target engagement and immunosupressive activities (to test proof of mechanism) | To determine whether blockade of ICOSL by AMG 557 leads to selective inhibition of the antigen-specific IgG antibody response (in keeping with the known biology of the ICOS/ICOSL costimulatory pathway) | To assess 'immunocompetency' |
| **2b. Describes rationale for a specific challenge regimen** | no | no | no | no | no | no | no |
| **2b: detail (what was the rationale for the specific challenge regimen)** | NA | NA | NA | NA | NA | NA | NA |
| **3a. Describes KLH form** | yes | no | yes | no | yes | yes | yes |
| **3b. Describes KLH product name** | yes | no | yes | no | yes | yes | yes |
| **3c. Describes KLH supplier/company** | yes | no | yes | no | yes | yes | yes |
| **3d. Describes adjuvant generic name** | NA | NA | NA | NA | NA | NA | yes |
| **3e. Describes adjuvant product name** | NA | NA | NA | NA | NA | NA | yes |
| **3f. Describes adjuvant supplier** | NA | NA | NA | NA | NA | NA | yes |
| **4a. Describe KLH preparation procedure** | no | no | no | no | no | no | yes |
| **4b. Describe KLH route of administration** | yes | no | yes | no | yes | yes | yes |
| **4c. Describe KLH site of administration** | no | no | no | no | no | no | no |
| **4d. Describe rechallenge route of administration** | yes | yes | NA | NA | NA | yes | NA |
| **4e. Describe rechallenge site of administration** | no | no | NA | NA | NA | no | NA |
| **4f. Describe KLH administration device (e.g. 27g needle)** | no | no | no | no | no | no | no |
| **5a. Describes who administered the challenge agent** | no | no | no | no | no | no | no |
| **7. Describes where the study was conducted** | no | yes | yes | yes | yes | yes | yes |
| **8a. Describes primary KLH dose** | yes | yes | yes | no | yes | yes | yes |
| **8b. Describes primary KLH timepoint relative to other study interventions** | yes | yes | yes | yes | yes | yes | yes |
| **8c. Describes rechallenge KLH doses** | yes | no | NA | NA | NA | yes | NA |
| **8d. Describes rechallenge KLH timepoints** | yes | yes | NA | NA | NA | yes | NA |
| **8e. Describes adjuvant dose** | NA | NA | NA | NA | NA | NA | no |
| **9. Describes use of KLH challenge in title** | no | no | no | no | no | yes | no |
| **10a. Describes use of KLH challenge in abstract** | yes | no | no | yes | yes | yes | no |
| **10b. Refers to assessment of immune response of an administered antigen in abstract** | yes | yes | yes | yes | yes | yes | no |
| **Blinding: were assessors of KLH DTH clinical response blinded to dose level or placebo (NA if no placebo, single dose level, or if no DTH performed)** | NA | no | NA | NA | NA | NA | NA |
| **Method of KLH challenge is referenced (i.e. reference to a KLH protocol published elsewhere)** | no | no | no | no | no | no | no |

**Consensus TIDieR checklist for identified early phase clinical trials utilising KLH challenge (n=14) – continued**

| **lead_author** | **Karnell** | **Espié** | **Yang** | **Saghari** | **Bingham** | **Van der Kolk** | **Smith** |
| --- | --- | --- | --- | --- | --- | --- | --- |
| **title** | A CD40L-targeting protein reduces autoantibodies and improves disease activity in patients with autoimmunity | First-in-human clinical trial to assess pharmacokinetics, pharmacodynamics, safety, and tolerability of iscalimab, an anti-CD40 monoclonal antibody | First-in-human study of the safety, tolerability, pharmacokinetics, and pharmacodynamics of ALPN-101, a dual CD28/ICOS antagonist, in healthy adult subjects | OX40L Inhibition Suppresses KLH-driven Immune Responses in Healthy Volunteers: A Randomized Controlled Trial Demonstrating Proof-of-Pharmacology for KY1005 | Immunization responses in rheumatoid arthritis patients treated with rituximab: results from a controlled clinical trial | Rituximab treatment results in impaired secondary humoral immune responsiveness | Treatment with Recombinant Growth Hormone Is Associated with Modest Improvement in CD4 Lymphocyte Reconstitution in HIV-Infected Persons on Antiretroviral Therapy: Results of ACTG A5174 |
| **2a. Describes rationale for using KLH challenge** | yes | yes | yes | yes | yes | yes | yes |
| **2a detail (what was the rationale for using KLH challenge)** | To evalute the ability of VIB4920 to influence hunmoral immune reponses in healthy participants | Pharmacodynamic assessment using T-cell dependent KLH immunization as a CD40-pathway-dependent tissue PD readout | To evaluate inhibition of T-cell dependent antibody responses | To explore PD effects of KY1005, including anti-KLH antibody titres and skin challenge response following ID KLH administration, seeking proof-of-pharmacology | To examine immunization responses (humoral and cellular immunity) in RA patients receiving rituximab | To examine immunization response in lymphoma patients | To evaluate cell-mediated response to antigens in HIV patients treated with rHGH |
| **2b. Describes rationale for a specific challenge regimen** | no | yes-partial | no | yes | no | yes | no |
| **2b: detail (what was the rationale for the specific challenge regimen)** | NA | Timepoints of KLH rechallenge based on predicted loss of CD40 receptor occupancy | NA | precident: previous study used this regimen | NA | described timing of KLH relative to study drug | NA |
| **3a. Describes KLH form** | no | no | no | yes | yes | yes | yes |
| **3b. Describes KLH product name** | no | no | no | yes | no | no | yes |
| **3c. Describes KLH supplier/company** | no | no | yes | yes | yes | yes | yes |
| **3d. Describes adjuvant generic name** | NA | yes | NA | yes | NA | NA | NA |
| **3e. Describes adjuvant product name** | NA | no | NA | yes | NA | NA | NA |
| **3f. Describes adjuvant supplier** | NA | no | NA | yes | NA | NA | NA |
| **4a. Describe KLH preparation procedure** | no | no | no | yes | no | yes | no |
| **4b. Describe KLH route of administration** | yes | yes | yes | yes | yes | yes | no |
| **4c. Describe KLH site of administration** | no | no | no | yes | no | no | yes |
| **4d. Describe rechallenge route of administration** | yes | yes | NA | yes | NA | NA | yes |
| **4e. Describe rechallenge site of administration** | no | no | NA | yes | NA | NA | yes |
| **4f. Describe KLH administration device (e.g. 27g needle)** | no | no | no | no | no | no | no |
| **5a. Describes who administered the challenge agent** | no | no | no | no | no | no | no |
| **7. Describes where the study was conducted** | no | yes | yes | yes | no | no | no |
| **8a. Describes primary KLH dose** | yes | yes | yes | yes | yes | yes | yes |
| **8b. Describes primary KLH timepoint relative to other study interventions** | yes | yes | yes | yes | yes | yes | yes |
| **8c. Describes rechallenge KLH doses** | yes | yes | NA | yes | NA | NA | yes |
| **8d. Describes rechallenge KLH timepoints** | yes | yes | NA | yes | NA | yes | yes |
| **8e. Describes adjuvant dose** | NA | no | NA | yes | NA | NA | NA |
| **9. Describes use of KLH challenge in title** | no | no | no | yes | no | no | no |
| **10a. Describes use of KLH challenge in abstract** | yes | yes | yes | yes | yes | no | no |
| **10b. Refers to assessment of immune response of an administered antigen in abstract** | yes | yes | yes | yes | yes | yes | no |
| **Blinding: were assessors of KLH DTH clinical response blinded to dose level or placebo (NA if no placebo, single dose level, or if no DTH performed)** | NA | NA | NA | yes | NA | NA | NA |
| **Method of KLH challenge is referenced (i.e. reference to a KLH protocol published elsewhere)** | no | no | no | yes | no | yes | no |
